# The view tolerance of human identity recognition depends on horizontal face information

**DOI:** 10.7554/eLife.108495

**Published:** 2026-07-20

**Authors:** Alexia Roux-Sibilon, Helene Dumont, Vincent Bremhorst, Christianne Jacobs, Valerie Goffaux

**Affiliations:** 1 Psychological Sciences Research Institute (IPSY), UCLouvain Louvain-la-Neuve Belgium; 2 Université Clermont-Auvergne, CNRS, LAPSCO Clermont-Ferrand France; 3 Statistical Methodology and Computing Service (SMCS), UCLouvain Louvain-la-Neuve Belgium; 4 Institute of Neurosciences (IoNS), UCLouvain Louvain-la-Neuve Belgium; University College London United Kingdom; University of Pennsylvania United States

**Keywords:** human face, identity recognition, view tolerance, model observer, horizontal tuning, Human

## Abstract

This study investigates which visual information enables humans to recognize facial identity across different viewpoints, a key unresolved question in vision science. Participants completed an identity recognition task using faces rotated across a range of yaw angles and filtered to retain specific orientation ranges of visual information. Regardless of viewpoint, human performance consistently relied on horizontal facial information. To understand why, we used model observers to assess the identity information physically available in the images. A view-selective model, which matched identities within the same viewpoint, indicated that diagnostic identity cues shift from predominantly horizontal in frontal views to more vertical in profile views. In contrast, a view-tolerant model, which matched identities across different viewpoints, revealed that horizontal information provides the most stable and reliable identity cues across views. Furthermore, horizontal facial information best predicted the average appearance of a face across viewpoints, supporting its role in forming stable identity representations. These findings suggest that view-tolerant face representations are acquired through exposure to the stable statistical properties of faces primarily conveyed by horizontal information. By specifying the spatial information underlying recognition across viewpoints, the study offers valuable empirical constraints for the development of theoretical and computational models of face recognition.

## Introduction

The way in which we recognize objects and people is a central but as yet unresolved question in vision science. Our understanding of visual perception is limited by our ability to account for how we recognize objects and people despite the sometimes radically different images they project onto the retinae, due to varying lighting, distance, depth rotation, etc. (DiCarlo and Cox, 2007). We experience faces under a broad range of depth rotations mainly along the x-axis, that is from left to right profile (i.e. yaw; Favelle and Palmisano, 2012) due to biomechanical constraints favoring x-axis head rotations and the vantage point on our conspecifics’ faces also varying more along the x than the y-axis (i.e. when moving around people). While rotating in depth, a given face projects retinal images that are more dissimilar than the ones that different faces under the same viewpoint would project (Adini et al., 1997; Burton et al., 2016; Hill et al., 1997). Yet, humans generally have no difficulty in recognizing a familiar face across views, which implies the joint ability to differentiate its identity from others and to generalize it from one view to another, that is with tolerance to variations (Ritchie and Burton, 2017).

The tolerance of face identity recognition is stronger for familiar than unfamiliar faces (e.g., Favelle and Palmisano, 2018; Hancock et al., 2000; Hill et al., 1997; Jeffery et al., 2006; Johnston and Edmonds, 2009; Jones et al., 2017; Liu and Chaudhuri, 2002; Newell et al., 1999; O’Toole et al., 1998; Troje and Bülthoff, 1996), suggesting that a core determinant of tolerant recognition is the repeated exposure to the natural statistics of a person’s face (e.g. to the variability of a person’s appearance across different views; Tian and Grill-Spector, 2015; Troje and Bülthoff, 1996; Van Meel and Op de Beeck, 2018; Wallis and Bülthoff, 2001). Such statistical learning is assumed to be the main unsupervised learning route for tolerant face/object recognition in humans and animals (DiCarlo and Cox, 2007; Fiser and Aslin, 2002; Hauser et al., 2001; Huber et al., 2023; Li and DiCarlo, 2008; Li and DiCarlo, 2010; Li and Dicarlo, 2012; Tian and Grill-Spector, 2015). Since seeing the different views of a given person in succession improves the tolerance of identity recognition, temporal contiguity is presumed to contribute to the statistical learning of face identity (Li and DiCarlo, 2010; Miyashita, 1993; Pitts and McCulloch, 1947; Van Meel and Op de Beeck, 2018; Wallis et al., 2009; Wallis and Bülthoff, 1999).

**Figure 1.**
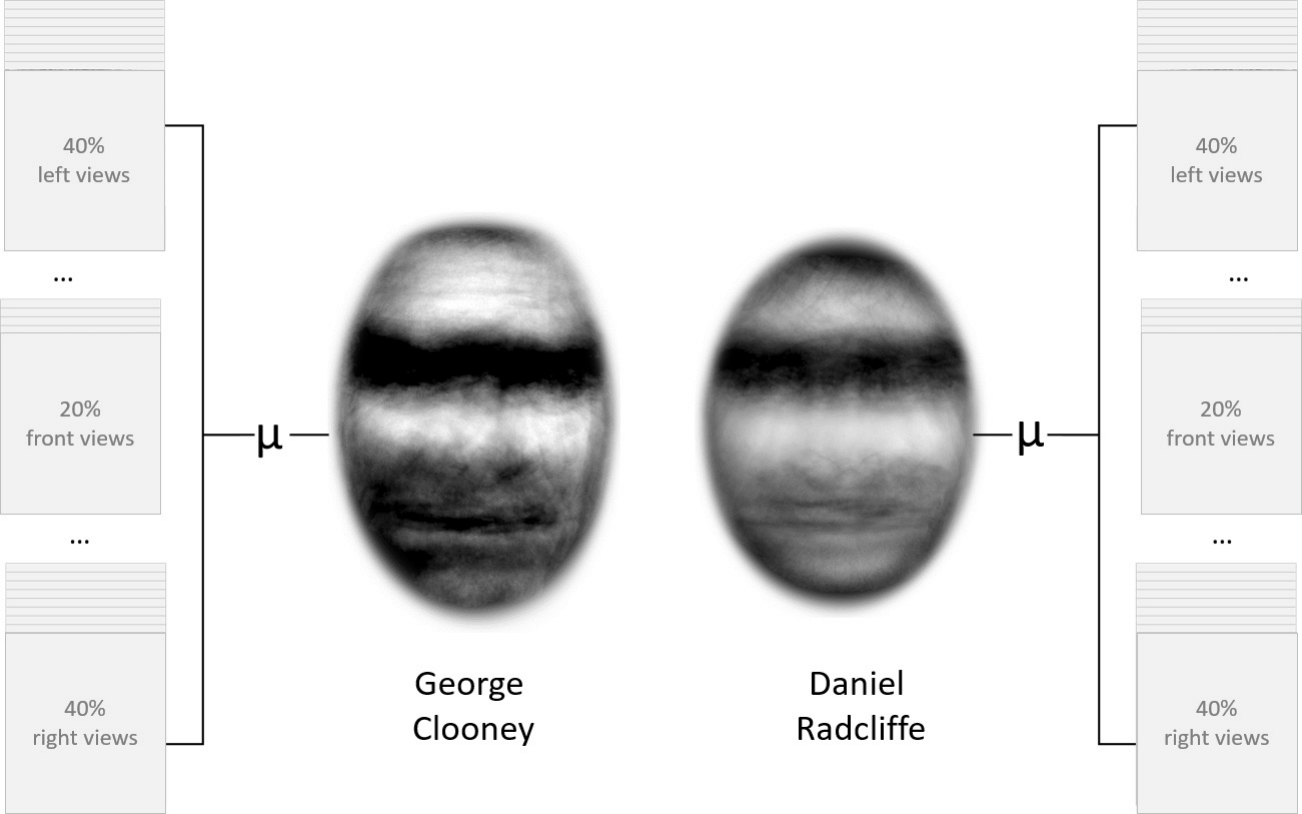
A graphic illustration of the horizontal structure that emerges from the average of multiple views of a face. The tolerance of human face identity recognition to drastic appearance variations caused by varying lighting, viewpoint, facial expression, etc. has been proposed to emerge through averaging (Burton et al., 2005). With increased exposure to a face, an averaging mechanism would progressively whiten accidental variations in appearance while preserving stable cues to identity. Past illustrations (Mike Burton, 2013; Burton et al., 2005) used varying lighting and expressions but moderate pose variations. Here, we show that averaging highly diverse views (from left to right profile) of a face produces a horizontally smeared image, which suggests that, across encounters with a face, cues at orientations other than horizontal are whitened. Images of two celebrities (George Clooney and Daniel Radcliffe) were sampled from the internet. Image averages were made of 40% left-averted, 40% right-averted, and 20% frontal views, in line with exposure to face views in natural viewing (Oruc et al., 2019). The luminance and RMS contrast of the averaged faces were set to a luminance of 0.5 and contrast of 0.4. Using this procedure, one can appreciate the emergence of the so-called bar code, namely, the vertical arrangement of horizontally oriented cues which carries the natural statistics of the face category and of face individual identity (Dakin and Watt, 2009).

While some attention has been devoted to the contribution of temporal contiguity in view-tolerant recognition, the *spatial* aspects of the natural statistics supporting view-tolerant face identity recognition are still largely elusive. Face appearance results from the complex interaction between extrinsic viewing conditions and the intrinsic 3D shape and reflectance properties of the face (determined e.g. by viewpoint and lighting; Favelle et al., 2017; Hill and Bruce, 1996; Liu et al., 2000). Burton and colleagues (Mike Burton, 2013; Burton et al., 2005) suggested that as exposure to multiple appearances of a person increases, the accidental, irrelevant variations would be progressively whitened (i.e. averaged out) and reveal the stable cues to identity. Figure 1 simulates such averaging using the pictures of a given identity taken from variable poses, as experienced in natural viewing (Oruc et al., 2019). It can be seen that additionally to the whitening of accidental properties, the resulting average contains a strong horizontal structure; namely, it results in the emergence of the so-called (horizontal) bar code of the face (Dakin and Watt, 2009).

We, and others, proposed that the horizontal content of the face stimulus may drive the visual mechanisms engaged for the view-tolerant recognition of face identity (Caldara and Seghier, 2009; Dakin and Watt, 2009; Gilad-Gutnick et al., 2018; Goffaux, 2008; Goffaux and Dakin, 2010). Several lines of empirical evidence indicate that the horizontally-oriented face information conveys optimal cues to identity. First, the visual mechanisms specialized for the identity recognition of faces’ frontal view rely preferentially on the horizontal structure of the face image, indicating a better sensitivity to identity in the horizontal range of face information (e.g. Balas et al., 2015; Dumont et al., 2024; Goffaux, 2019; Goffaux and Dakin, 2010; Pachai et al., 2013; Pachai et al., 2018). The horizontal dependence of face identity recognition has also been shown to predict face identification accuracy at the individual observer level (Duncan et al., 2019; Pachai et al., 2013). Furthermore, there is indirect evidence that horizontal face information may optimally drive the tolerance of face identity recognition. For example, while the horizontal dependence manifests from three months of age (de Heering et al., 2016), it strengthens over the lifespan, that is as individuals accumulate experience with the natural statistics of face appearance (Goffaux et al., 2015). Moreover, and considering that the recognition of familiar faces differs from unfamiliar face recognition by its stronger tolerance to retinal variations due to, for example a change in view (Bruce et al., 1999; Burton et al., 1999; Burton et al., 2010; Hill and Bruce, 1996; Megreya and Burton, 2006; O’Toole et al., 1998; Ramon, 2015), the finding by Pachai and colleagues that the recognition of a face from ¾ to full-frontal view increasingly relies on horizontal information as a function of familiarity provides indirect support to the notion that the tolerance of identity recognition most crucially depends on horizontal face cues (Pachai et al., 2017). In one of Goffaux and Dakin, 2010’s experiments, the matching of unfamiliar faces from frontal to ¾ view was more largely disrupted by horizontal than vertical noise masking. It is not clear, however, whether this effect reflects view-tolerant recognition or the horizontally tuned encoding of the frontal view probe. The important question of whether the horizontal range of face information is a privileged informational avenue for view-tolerant identity recognition is therefore still unanswered.

The present study aims at directly investigating the hypothesis that the view tolerance of human face identity recognition is supported by the horizontal range of face information. First, we familiarized a sample of human observers with multiple views of a set of face identities. In an old-new recognition task involving face stimuli presented in various views, we demonstrated that humans stay broadly tuned to the horizontal range of face information irrespective of yaw, with a stronger tuning observed for frontal views.

Second, we used a model observer approach to define the information physically available in the stimulus for the matching of face identity within a given viewpoint and across different viewpoints. A model observer is a basic image processing algorithm that cross-correlates a target image with probe images at the pixel level and selects the probe with the highest correlation, that is the most likely match. Combined with orientation filtering, this method provides a formal way to describe how the information most useful for matching identity is distributed in the orientation domain of the face image (e.g. Collin et al., 2014; Gold et al., 1999; Näsänen, 1999; for a similar approach in the spatial frequency domain). The pixelwise image correlation performed by a model observer is not taken as a computationally valid analog of human face processing; on the contrary, it is a means to separate information objectively available in the stimulus from human vision (Collin et al., 2014).

We tested two model observers on the same multiple views of face identities as used in the human old-new recognition task. One so-called *view-tolerant* model observer matched identities across distinct views (e.g. matching a profile view of identity A with the other views of all identities). This model observer necessarily relied on the physical image properties that were most stable across views. We compared the performance of the view-tolerant model to a *view-selective* model observer, which matched identities within the same view (e.g. matching a profile view of identity A with the profile view of all identities), therefore revealing what orientations were objectively (irrespective of human vision) the most diagnostic to match faces at each view specifically.

This approach revealed a substantial difference in the orientation distribution of view-specific and view-tolerant information. The view-selective model indicated that, at the image level, the orientation ranges supporting view-selective identity matching differ drastically from one view to another: identity cues were distributed in the horizontal range at frontal views (in line with Goffaux, 2019; Keil, 2008; Pachai et al., 2013) but shifted towards the vertical range as the face turned to profile. In contrast, the view-tolerant model performance indicated that the horizontal structure of the face stimulus provides the most stable cues for matching identity across yaws. Partial correlations between model and human observer performance suggest that the horizontal tuning of human face identity recognition is due not only to the high diagnosticity of horizontal information at frontal view but also to the stability of the horizontal identity cues across views.

## Results

### Human observers

**Figure 2.**
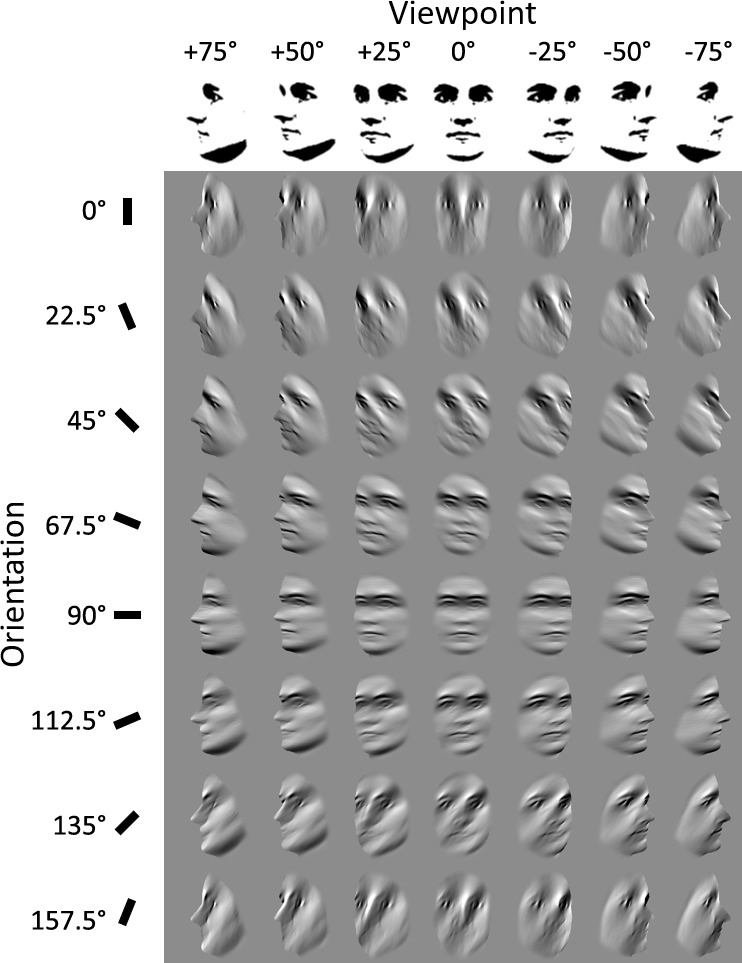
Stimulus conditions. *Columns*. Each identity was viewed from seven different viewpoints ranging from +75° to –75° in steps of 25°. *Rows*. All images were filtered in the Fourier domain to preserve only a selective range of orientation, from 0° (vertical) to 157.5° in steps of 22.5°.

Human face identification performance was measured using an old/new recognition task. The stimuli consisted of unfamiliar 3D laser-scanned faces (Troje and Bülthoff, 1996) captured from seven viewpoints ranging from –75° (left profile) to +75° (right profile) in 25° increments (full-face views at 0°; see Figure 2). Twenty-two healthy young adults were familiarized with the full-spectrum version of half the identities before performing the old/new recognition task on the complete set of stimuli filtered in the Fourier domain to preserve energy at selective orientations ranging from 0° (vertical) to 157.5° in steps of 22.5°.

**Figure 3.**
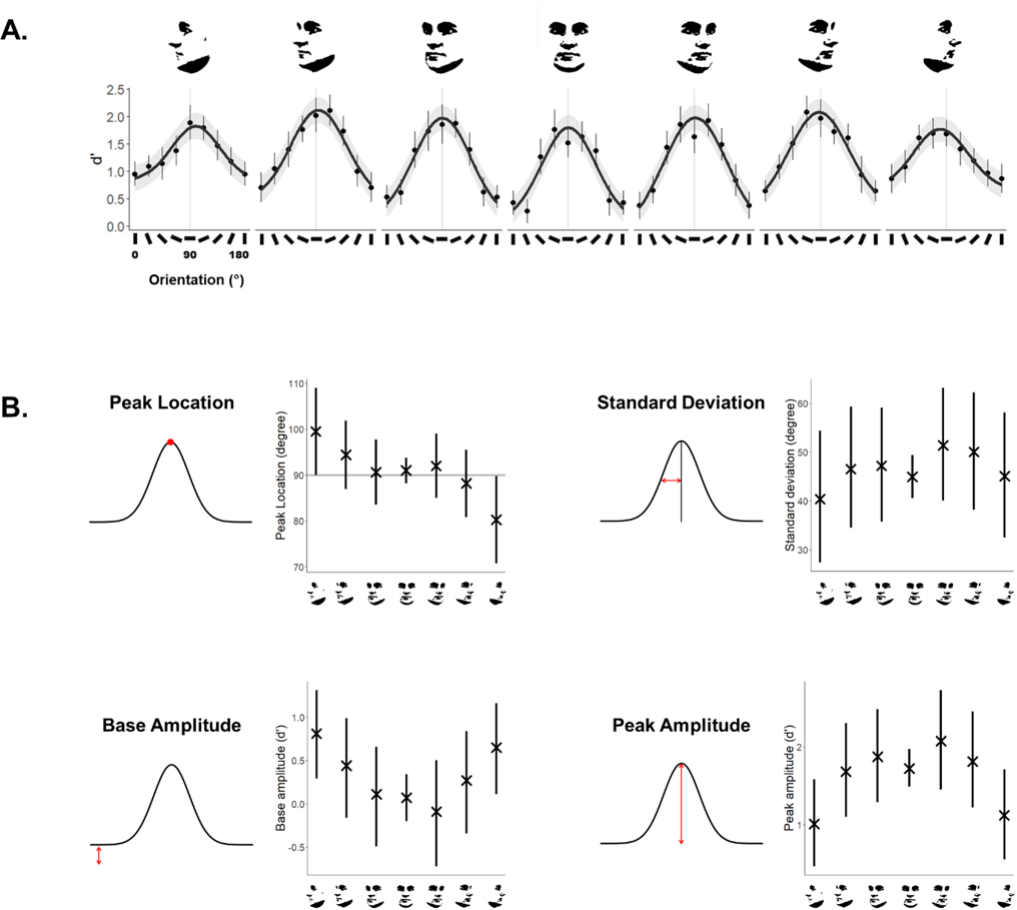
Human face identity recognition: orientation tuning across viewpoint. (**A**) Sensitivity of human observers (n = 22) to facial identity (d’) as a function of the orientation filter (0° to 180° in 22.5° steps), and face viewpoint (yaw: +75° to –75° in 25° steps). Dots and error bars represent mean *d’* values and 95% confidence intervals across participants. Solid lines and shaded areas indicate the mean posterior predictions and 95% credible intervals from the Gaussian Bayesian multilevel model. (**B**) Population-level mean parameters of the Gaussian Bayesian Multilevel model: peak location, standard deviation, base amplitude, and peak amplitude. Each estimate is plotted with 95% credible intervals as a function of face viewpoint. The 95% credible intervals reflect the uncertainty of the model. They indicate a 95% probability that the true population parameter lies within the specified range, given the observed data.

Upon visual inspection, the group-averaged recognition d’ plotted as a function of orientation showed a bell-shaped curve roughly centered on the horizontal orientation in the frontal view condition, as well as in the other viewpoint conditions (Figure 3A). The fitting confirmed that sensitivity to identity shows a roughly similar Gaussian orientation tuning profile across viewpoints (see bell-shaped curves on Figure 3A; Table 1).

**Table 1.** Posterior mean and 95% credible interval for each parameter of the Gaussian model, at each viewpoint.

Parameter	Viewpoint	Estimate (posterior mean)	95% Credible interval – lower bound	95% Credible interval – upper bound
Peak location	–75 (left profile)	99.5	90.02	108.98
	–50	94.4	86.98	101.87
	–25	90.62	83.58	97.73
	0 (full front view)	90.99	88.22	93.78
	25	91.98	85.00	99.01
	50	88.19	80.82	95.51
	75 (right profile)	80.24	70.73	89.76
Standard Deviation	–75 (left profile)	40.37	27.40	54.4
	–50	46.51	34.51	59.29
	–25	47.2	35.75	59.07
	0 (full front view)	44.94	40.55	49.41
	25	51.36	40.04	63.14
	50	50.02	38.18	62.23
	75 (right profile)	45.06	32.5	58.12
Base amplitude	–75 (left profile)	0.81	0.29	1.31
	–50	0.44	–0.16	0.99
	–25	0.11	–0.49	0.66
	0 (full front view)	0.07	–0.2	0.34
	25	–0.09	–0.72	0.5
	50	0.27	–0.34	0.84
	75 (right profile)	0.65	0.11	1.16
Peak amplitude	–75 (left profile)	1.01	0.47	1.58
	–50	1.68	1.10	2.30
	–25	1.87	1.29	2.48
	0 (full front view)	1.72	1.49	1.97
	25	2.07	1.45	2.72
	50	1.81	1.22	2.45
	75 (right profile)	1.12	0.56	1.71

**Figure 4.**
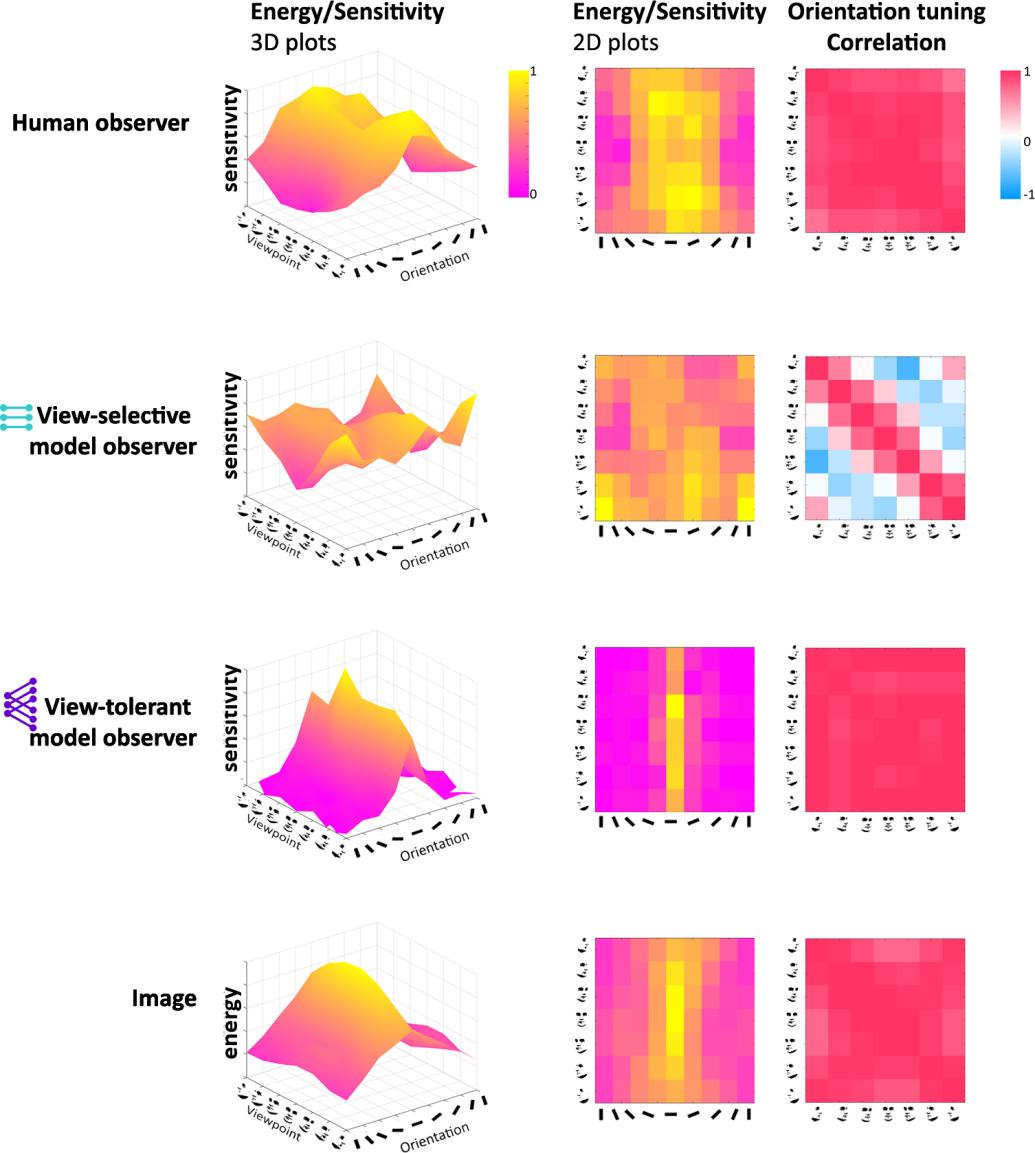
Sensitivity (i.e. performance in the recognition task) of human (n = 22) and model observers (n = 22 each) and image energy across viewpoints and orientations. *Left column*. 3D surf plots of the normalized energy/sensitivity across orientation and viewpoints. *Middle column*. Matrix representations of the normalized energy/sensitivity across orientation and viewpoints. *Right column*. Matrix representations of the Pearson correlation (non-Fisher Z-transformed) of the normalized orientation distributions of energy/sensitivity across viewpoints.

The relative stability of the human orientation sensitivity profile was corroborated by the stable and significantly positive correlations of the orientation tuning profile across viewpoints (rs >0.67, ps <0.05; Figure 4).

**Figure 4—figure supplement 1.**
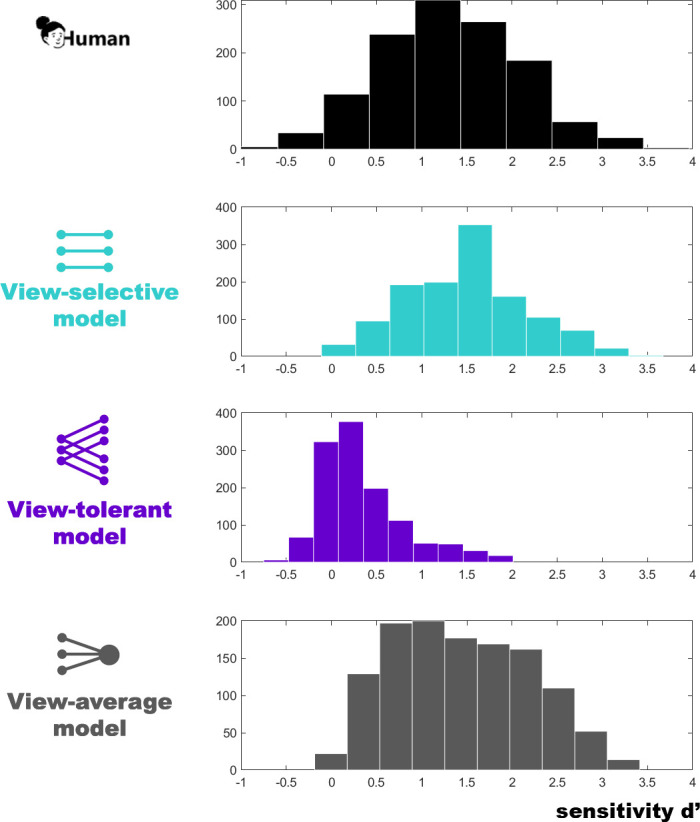
Distribution of sensitivity d’ of human, view-selective model, view-tolerant model, and view-average model observers, all viewpoint and orientation conditions confounded.

While human sensitivity for face identity was best around horizontal orientations and worse around vertical ones across viewpoints, there were notable fluctuations in the orientation sensitivity profile. To better grasp these variations, we plotted the population-level estimates of the four parameters of the Gaussian curve as a function of viewpoint in Figure 3B. Population-level estimates and 95% credible intervals can also be found in Table 2.

**Table 2.** Mean and 95% confidence interval of the (Fisher Z-transformed) partial correlation coefficients between human and each model orientation *d’* profiles while controlling for the variance in image energy and alternate model.

Model	Viewpoint-specific		Viewpoint-tolerant	
	Mean	95% CI	Mean	95% CI
+075	0.06	[–0.09 0.2]	0.49	[0.30 0.67]
+050	0.04	[–0.10 0.18]	0.5	[0.34 0.66]
+025	0.56	[0.42 0.71]	0.3	[0.17 0.42]
000	0.79	[0.62 0.97]	0.19	[0.09 0.30]
–025	0.36	[0.18 0.54]	0.34	[0.17 0.51]
–050	0.1	[–0.06 0.25]	0.59	[0.45 0.72]
–075	–0.05	[–0.17 0.08]	0.56	[0.35 0.77]

*Peak location* is estimated to lie close to 90° (grey horizontal line in Figure 3B) at all viewpoints except at the two profile views where peak location shifts toward adjacent oblique orientations (yaw = –75/75; see Figure 3A). Specifically, peak human sensitivity tended to shift towards left and right obliques for the most extreme leftward and rightward deviations in viewpoint, respectively. *Peak amplitude*, which corresponds to the difference in sensitivity between the vertical and horizontal ranges, is relatively stable across all viewpoints except for the two profile views where it is lower. *Base amplitude*, reflecting sensitivity in the vertical range, is highest for the two profile views and progressively decreases towards the full-frontal view. This pattern suggests that vertically oriented face content is more diagnostic for profile than for other viewpoints. The variation of *Standard Deviation* across viewpoints can hardly be interpreted because of the high uncertainty of the estimations; the 95% credible intervals of the *Standard Deviation* for the different viewpoints mostly overlap.

### View-selective model observer

The performance of the model observer matching faces at specific viewpoints indicates that the most objectively diagnostic orientation range varies as a function of viewpoint (Figure 4).

Within the frontal view, optimal cues to identity are conveyed by orientations close to the horizontal angle (between 90° and 112.5°). The striking similarity of the orientation tuning profile of the view-selective model observer with the human performance indicates that at frontal view, the human visual system makes an efficient use of the information physically available in the stimulus. The peak of model performance shifted to the left and right oblique orientations closest to horizontal angle (67.5° and 112.5°) for leftward and rightward deviations in viewpoint, respectively. Additionally, the view-selective model observer increasingly relied on vertical cues as the face is turned towards its profile, in line with human performance.

We only found weak (but significant) positive correlations of the orientation sensitivity profiles across adjacent viewpoints (e.g. +075 and+050) confirming the orientation tuning variations across views (Figure 4).

Except for the frontal view, the orientation tuning of the view-selective model observer differs from human orientation profiles, which kept tuned to horizontal and adjacent oblique ranges irrespective of viewpoint.

### View-tolerant model observer

The model observer matching face identity across viewpoints performed in a drastically different way from the view-selective model observer. Recognition performance peaked sharply in horizontal angles and dropped abruptly at other ranges (Figure 4). Such orientation profile was relatively stable across viewpoints, as shown by the homogeneous matrix of large positive and significant Pearson correlation coefficients for sensitivity profiles across viewpoints (rs >0.88, ps <0.002).

Such horizontal tuning was sharper than the one observed for humans, with a much more severe drop of sensitivity in the vertical orientation range. The horizontal tuning of human performance is much shallower even at frontal view. It shows a similar dip in sensitivity for the vertical range but tends to decrease when moving away from the frontal view, which does not happen for the view-tolerant model observer that keeps sharply horizontally-tuned.

### Human versus model observers

What model observer best predicts the orientation tuning profile of human face recognition? Is it the view-selective model taking advantage of the identity cues that are optimal at selective viewpoints, or the view-tolerant model, which relies on the identity cues that are the most stable across viewpoints? To address this question directly, human orientation *d’* profiles were correlated at the individual level with each model orientation *d’* profile, when partialling out the variance explained by the alternate model and image energy. We submitted the so-obtained individual Fisher Z-transformed partial correlation coefficients to a repeated-measure ANOVA with Model and Viewpoint as within-subject factors.

**Figure 5.**
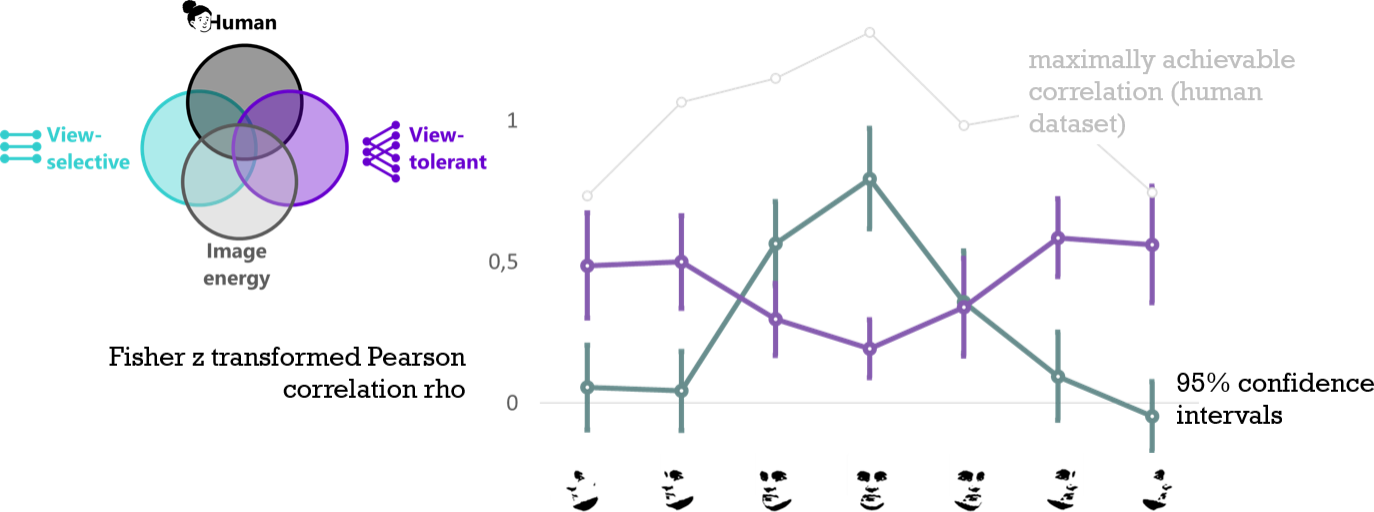
Fisher Z-transformed Pearson partial correlation of the orientation sensitivity profiles between humans and each model (n = 22), while controlling for the alternate model and image energy profiles. Error bars show the 95% confidence intervals. The faded gray line depicts the maximally achievable correlation for the separate viewpoint conditions in the human dataset (see Methods for details).

These partial correlations showed that the orientation tuning profiles of human and view-selective model observers correlated strongly for frontal and near-frontal views. However, partial correlations dropped steeply as the views deviated further from frontal (Figure 5; Table 2). The correlation of orientation tuning profiles between human and view-tolerant model observers was lower overall but significant across all viewpoints.

The repeated-measure ANOVA confirmed that the partial correlations between human and model orientation sensitivity profiles differed depending on Model (F(1,21)=5.21, p=0.033, η²=0.017) and Viewpoint (F(3.85,80.9)=11.064, p<0.001, η²=0.11). The interaction between Model and Viewpoint was also significant (F(4.2,88.35)=13.035, p<0.001, η²=0.23).

We explored the impact of viewpoint on human-model correlation for the view-selective and view-tolerant models separately using a repeated-measure ANOVA with Viewpoint as a within-subject factor. For the view-selective model, this analysis revealed a robust effect of Viewpoint on the human-model partial correlation (F(3.86,80.99)=22.795, p<0.001, η²=0.52). For the view-tolerant model, the effect of Viewpoint on the human-model partial correlation was not significant (F(6,126)=1.2, p=0.32, η²=0.054). This confirms the relatively stable human-model partial correlation coefficients across viewpoints observed for the view-tolerant model and the fluctuant profile of human-model partial correlation coefficients for the view-specific model (peaking at frontal views and decreasing as moving toward profile views).

Furthermore, at each viewpoint, we examined which model observer best correlated with the human orientation sensitivity profile (using Holm-corrected post-hoc tests on Fisher Z-transformed partial correlation coefficients; Table 3). The view-tolerant model predicted a significantly larger portion of the variance in human orientation sensitivity profile at ±050 and ±075 viewpoints; this difference was marginal for the +050 viewpoint. It is only for the identification of frontal views of faces that the view-selective model correlated best with human data. Correlations were of similar strength for the +075, +025, –025 (and +050) viewpoints (Table 3).

**Table 3.** Difference of the human-model partial correlation coefficients between viewpoint-selective and view-tolerant model observers. Positive t values indicate a stronger correlation with the viewpoint-selective model, and negative t values indicate a stronger correlation with the view-tolerant model.

		95% CI for mean difference			
View	Mean difference	Lower	Upper	t	p Holm
+075	–0.47	–0.86	–0.07	–4.20	0.002
+050	–0.43	–0.82	–0.03	–3.82	0.01
+025	0.23	–0.16	0.63	2.08	1
+000	0.69	0.30	1.09	6.21	<.001
–025	0.03	–0.37	0.42	0.23	1
–050	–0.61	–1.00	–0.21	–5.44	<.001
–075	–0.78	–1.18	–0.39	–7.02	<.001

### Is model observers’ performance predicted by horizontal energy predominance?

In the above correlation analyses, we controlled the variance in image energy to yield a clean measure of the functional link between human and model observer performance. Here, we explore the possibility that the horizontal reliance of either model observer performance scales with the energy predominance of this orientation range in the stimulus image.

To analyze the influence of horizontal predominance on the model’s performance, we computed for each image the horizontal minus vertical difference in terms of energy and model observer sensitivity. For each identity and at each viewpoint, energy and model sensitivity difference values were submitted to a Pearson correlation. We found a modest positive correlation between view-selective model observer and image energy (Pearson *r*=0.24, p<0.0005). There was no similar correlation for the view-tolerant model (Pearson rho=0.025, p=0.72) despite energy and view-tolerant model performance similarly peaking in the horizontal range across viewpoints.

What this analysis shows is that, in a given face, the stronger the predominance in horizontal energy (relative to the vertical energy), the more diagnostic the horizontally oriented identity cues for view-selective recognition. However, horizontal energy predominance in a given face does not account for this range carrying the most stable cues across viewpoints.

This image-level analysis did not include human data since human sensitivity necessarily aggregates performance across trials. However, it would likely show a similar detachment from horizontal energy predominance as the view-tolerant model. Namely, humans are expected to rely on horizontal cues to match faces across views no matter the amount of horizontal energy predominance in the face at stake (e.g. George Clooney versus Brad Pitt).

## Discussion

Tolerant face identity recognition relies on the ability to extract the idiosyncratic identity cues of a face from its highly variable appearance (e.g. Burton et al., 2016; Kramer et al., 2018). The spatial information supporting the tolerance of visual recognition is a central and debated topic in the field of visual and computational neurosciences (e.g. Andrews et al., 2023; DiCarlo and Cox, 2007). By showing that the information supporting tolerance in face identity recognition is image-computable, that is that it can be objectively defined in the orientation domain of the face image, the present work makes a decisive advance on this question. Our finding that view-tolerant face identity recognition is driven by the horizontal range of face information yields concrete, image-driven constraints for the development of theoretical and computational models of visual recognition.

Human participants performed an old/new identity recognition task on face stimuli presented under a variable view (ranging from left to right profile) and filtered to contain a restricted range of oriented content (ranging from 0° to 157.5° in steps of 22.5°; see Figure 2). For each view, recognition performance followed a Gaussian profile with a peak in the horizontal range and declining progressively when shifting towards the vertical range (Figure 3). Yet, while identity recognition stayed broadly tuned to the horizontal range irrespective of the vantage view, there were moderate but notable fluctuations in the tuning profiles. First, the peak location of the Gaussian tuning profile slightly and gradually shifted away from horizontal towards right and left adjacent obliques as the face turned to left or right profile, respectively. Second, the base amplitude of the Gaussian increased drastically towards profile views. The U-shaped profile of base amplitude as a function of viewpoint shows the increasing contribution of vertically oriented information as the face moves away from the frontal view (see Figure 3B). In other words, while human identity recognition stays tuned to the horizontal range irrespective of viewpoint, it tends to increasingly rely on vertical (and close-to-vertical) orientations as the face turns to profile.

As the vantage point of a face shifts away from frontal view, morphological features related to the 3D structure of the face become more apparent (e.g. nose and cheek protuberances, jaw line, nose bridge, eyebrow head; see e.g. Stephan and Caine, 2007 for evidence that the nose gains in informativeness from frontal to profile view). Our finding of moderate but systematic fluctuations in the tuning peak location and that vertical range gains importance in non-frontal views suggests that non-horizontal orientations may facilitate access to such features.

In contrast, other sources of information are lost when shifting towards profile views, such as the bilateral symmetric organization of the face as well as the 2D shape of features and their configuration along the x axis (e.g. size of eyebrows, interocular distance; Royer et al., 2016; Troje and Bülthoff, 1996). In a way, it is surprising that this shift in accessible information did not manifest in a more substantial variation of the orientation tuning peak location. Instead, the relatively stable preference for horizontal information across views suggests that it is not solely due to this range facilitating access to the 2D shape properties and bilateral symmetry (Dakin and Watt, 2009). It aligns with our recent evidence (Dumont et al., 2024) that the horizontal face information yields not only 2D-shape but also surface cues to identity. The stable horizontal tuning may also be due to identity recognition relying mostly on the eye region irrespective of view. Indeed, despite the eye appearance being strongly affected by changes in view, the identity information extracted from this region is the most diagnostic (Stephan and Caine, 2007; but see Royer et al., 2016 for conflicting results) and best defined in the horizontal range irrespective of view.

Using a model observer approach, we investigated how the human observer makes use of the identity cues physically available in the image across orientations and views. It is indeed important to compare the human tuning profile to an image-based quantification of the information that is available in the stimulus in order to characterize more formally the human sampling specificities at stake when recognizing face identity. We designed two model observers to measure the information available in the stimulus to match (i.e. cross-correlate) face identity within and across views and disentangle stimulus information available for view-selective and view-tolerant recognition, respectively.

Let’s first summarize the findings related to the view-selective model observer. It showed that the diagnostic orientation ranges for matching face identity in a view-specific manner vary greatly across views (Figure 4). The view-selective model observer was horizontally-tuned for frontal views of faces, in a manner strikingly similar to the human performance profile. This suggests that human observers make a close-to-optimal use of orientation information in face images when identifying frontal face views. Akin to human recognition, the view-selective model progressively increased its reliance on vertically-oriented cues when the face moved from frontal to profile. However—and in contrast to human performance—this came at the expense of the horizontal tuning, which vanished completely. What these findings suggest is that while the horizontal range conveys the optimal cues to identity at frontal view, it loses its informativeness in non-frontal views; at the image pixel level, face identity at non-frontal views is indeed predominantly carried by non-horizontal ranges of information. Therefore, view-specific informativeness does not account for the generalization of the horizontal tuning profile of human identity recognition across views.

In contrast to the view-selective model observer, the view-tolerant model observer kept sharply tuned to the horizontal range irrespective of view (Figure 4). The horizontal range resulted in the highest cross-correlation among the different views of a given face indicating that this range yields the identity cues that are the most stable across views, that is those that enable binding different face views into a unique representation of identity (Burton, 2013). This physical property of the face image likely explains why human identity recognition keeps horizontally tuned across views (see also Goffaux and Dakin, 2010).

For a direct comparison of human and model performance, we quantified the variance shared between each model observer and human orientation tuning profile while controlling image energy and the alternate model (Figure 5). These analyses confirmed that the view-selective model best predicted the orientation tuning of human identity recognition at frontal and close-to-frontal views, which are typically experienced during face-to-face conversations, but not at profile and close-to-profile views. In contrast, the variance explained by the view-tolerant model was relatively stable across viewpoints. The particularly strong horizontal tuning of human identity recognition is thus likely due to the visual system extracting a representation that simultaneously prioritizes the orientation range conveying the cues that are the richest at frontal view and the most stable across views.

**Figure 6.**
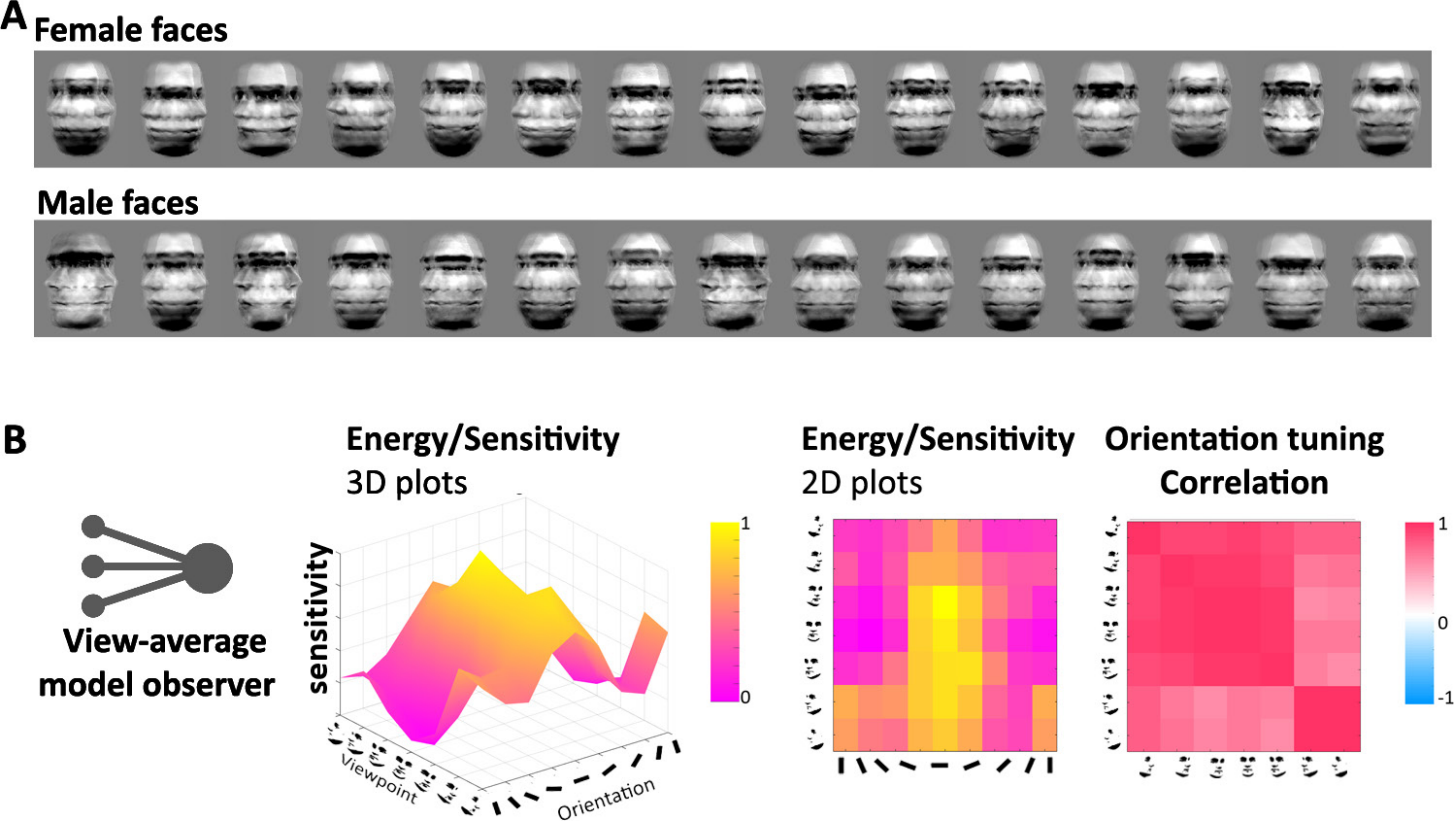
View-average model observer. (**A**) Image averages of the different views of each face identity of the stimulus set. (**B**) Recognition performance (normalized sensitivity d’) of the view-average model observer across views and orientations. *From left to right:* 3D surf plots of the normalized sensitivity across orientation and viewpoints, matrix representations of the normalized sensitivity across orientation and viewpoints, and matrix representations of the Pearson correlation of the normalized orientation distributions of sensitivity across viewpoints.

Tolerant representation of identity presumably builds up through repeated exposure to the physical variability of face appearance (Mike Burton, 2013; Burton et al., 2005). Over encounters, the idiosyncratic properties of a face are reinforced in its internal representation at the expense of uninformative accidental properties, the representation of which gradually attenuates (Figure 1; Tian and Grill-Spector, 2015; Troje and Bülthoff, 1996; Van Meel and Op de Beeck, 2018; Wallis and Bülthoff, 2001). The stable identity cues are horizontal, which entails that this range should best predict the average face appearance across views. Following a suggestion from a reviewer of this paper, we addressed this hypothesis by designing an additional model observer, which matched the experimental stimuli (i.e. orientation-filtered images of faces seen under different views) to the average of the different views of each face identity in full spectrum (Figure 6; see details in Appendix 1). In line with our hypothesis, this ‘view-average’ model observer performed best with horizontally filtered faces. These additional results support our suggestion that the average summary of a face identity contains a strong horizontal structure, a so-called (horizontal) bar code (Figure 1), which drives the view-tolerant recognition of face identity (Caldara and Seghier, 2009; Dakin and Watt, 2009; Gilad-Gutnick et al., 2018; Goffaux, 2008; Goffaux and Dakin, 2010).

Our stimuli, originally designed by Troje and Bülthoff, 1996, are 3D laser scans of white individuals aged between 20 and 40 years, posing with a neutral expression and shot under a fixed illumination. Ears and a small portion of the neck were visible while the hair region was removed. All face images had a normalized skin color, and we further converted them to grayscales. The use of such a controlled set restricted the range of within- and between-identity variations compared to what is experienced in natural settings. However, the skin color normalization and grayscale conversion, while limiting the range of face variability, did not eliminate the contribution of surface pigmentation in our study as the grayscale 3D laser scanned faces used here contained natural variations in crucial surface cues such as skin albedo and texture. For these reasons, we believe that the present findings generalize to more ecological viewing conditions.

The horizontal tuning of human face recognition was found to be relatively broad across views, compared with the sharp tuning of the view-tolerant model observer. The tuning breadth of human recognition may serve to retain the complex variability of face appearance across views. Indeed, each face identity has its own idiosyncratic way to vary across expression, illumination, view, etc. Such idiosyncratic within-person variability has been proposed to drive familiar face identity recognition as much as the stable (horizontal) identity information (e.g. Burton et al., 2016; Ritchie and Burton, 2017). Moreover, accidental properties such as head and gaze orientation carry important cues for the regulation of social interactions. Our past evidence shows that the fine discrimination of gaze direction is best supported in the vertical range (Goffaux, 2019). The vertical range also likely carries most of the information about the head direction. Thus, the broad horizontal tuning of identity recognition by humans may allow for the integration of such accidental properties of a face with the (more) stable identity (Or and Wilson, 2010). For functional social interactions, it may be advantageous to retain the dynamic and variable signals emitted by a face as much as its invariant aspects, which would entail a relatively broad tuning to orientation.

Effect of lighting is even more disruptive than view changes (Adini et al., 1997; Braje et al., 1998; Favelle et al., 2017; Hill and Bruce, 1996; Johnston et al., 2013; Tarr et al., 1998; Tarr et al., 2008). Image representations that emphasized the horizontal features were found to be less sensitive to changes in the direction of illumination (Adini et al., 1997). Future research should test whether tolerance of human face identity recognition to lighting is also supported by the horizontal range.

To conclude, this study demonstrates that the horizontal range carries the richest identity cues in frontal views, and the most stable across views. The orientation tuning profile of human identity recognition aligns with this combination of high diagnostic value in frontal views and cross-view stability. Taken together, this body of evidence suggests that the invariant representation of face identity, gradually learned through repeated exposure to its natural appearance statistics, relies heavily on horizontal facial information (Figure 1; Burton et al., 2016; Dakin and Watt, 2009; Ritchie and Burton, 2017).

## Materials and methods

### Subjects

Twenty-two healthy young adults took part in this experiment in exchange for monetary compensation (8 euros/hr of testing). They were 14 females and 8 males, aged 23.5 (±3.4) on average (4 were left-handed), recruited via Facebook advertisement. They received a written description of the experiment protocol and gave their written informed consent. Participants had normal vision as verified by a Landolt C acuity test (conducted using FRACT; Bach, 1996). Participants wore optical corrections when necessary. The experimental protocol was approved by the local ethical committee (Psychological Sciences Research Institute, UCLouvain).

### Stimuli

We selected 30 face identities (15 male, 15 female) from the 3D laser-scanned face database of the Max-Planck Institute for Biological Cybernetics (Tuebingen, Germany; Troje and Bülthoff, 1996). Faces were viewed under seven different viewpoints ranging from –75° (left profile) to +75° (right profile) in steps of 25° (full front faces at 0°; see Figure 2). We first converted all images to a gray scale ranging from 0 to 1 and resized them so that all faces subtended a height of 210 pixels. All images were padded into 400 × 400 pixels gray canvas and alpha-blended with a view-specific aperture designed to cover the hair and neck of the average of all face images at a given viewpoint (using Adobe Photoshop).

Next, images were normalized to obtain a mean luminance of 0 and root-mean square (RMS) contrast of 1 and submitted to a fast two-dimensional Fourier transform to manipulate orientation content. Since manipulations in the Fourier domain apply to the whole image, they encompass both the face and background pixels. When the image of a face on a plain background is filtered in the Fourier orientation domain, energy belonging to the face therefore smears to the background and vice-versa, resulting in an oriented halo. To minimize this smearing, we applied an iterative phase-scrambling procedure (as in Canoluk et al., 2023; Petras et al., 2019; Roux-Sibilon et al., 2023; Schuurmans et al., 2023), which consists in iteratively phase-scrambling the image, pasting the original face pixels, and phase-scrambling again (50 iterations). By making the power spectra of the face and background pixels more comparable, this procedure minimizes smearing during orientation filtering. The amplitude spectra of the resulting images were then multiplied with wrapped Gaussian filters (SD of 20°) centered on orientations ranging from 0° (vertical) to 157.5° in steps of 22.5°. After inverse-Fourier transform, the filtered images were combined with a view-specific aperture.

Images of faces cropped from their background as used here contain most of their energy in the horizontal range (Goffaux, 2019; Goffaux and Greenwood, 2016; Keil, 2009). Across yaws, we found face energy to range between 0.11 and 0.14 on a 0–1 grayscale, which is moderate compared to the range of face energy variations we measured across identities (from 0.08 to 0.18). To prevent energy from explaining our results, in all images, the luminance and RMS contrast of the face pixels were fixed to 0.55 and 0.15, respectively, and background pixels were uniformly set to 0.55. The percentage of clipped pixel values (below 0 or above 1) per image did not exceed 3%.

We used Matlab 2014a (Mathworks Inc, Natick, MA) for stimulus preparation. Stimuli were displayed against a gray background (0.55 luminance across RGB channels) at a viewing distance of 57 cm on a Viewpixx monitor (VPixx Technologies Inc, Saint-Bruno, Canada) with a resolution of 1920 × 1080 pixels and a 70 Hz refresh rate using PsychToolbox (Brainard, 1997). With this display, face area subtended 5° (width) by 8° (height) of visual angle (approximately corresponding to conversational distance). At the start of the experiment, lighting was switched off, and the testing area of the lab was closed off separately with light-draining black curtains.

### Procedure

Face identification performance was measured with an old/new recognition task.

Participants were familiarized with five female and five male faces. These familiar identities were randomly sampled from the stimulus set of 30 face identities on the participant’s first visit and were kept identical throughout the experiment. In the first testing session, we familiarized each participant with their selection of to-be-learnt ‘old’ faces, presented in their full-spectrum version. To engage participants in the face learning procedure, they were asked to remember a face–name pairing, although during the main experiment they were only asked to judge whether the face was seen during familiarization (‘old’) or new (i.e. incidental learning method as in Liu and Chaudhuri, 2002). Each identity was first shown centrally rotating from left profile (+75°) to right profile (–75°) pseudo-dynamically along with its assigned first name on top of the screen (400 ms per frame). The various views of the given face (in succession from +75° to –75° in steps of 25°, i.e. seven views) were then presented one by one for 400 ms. After each identity presentation, a recap screen appeared with all the faces learned in previous trials, clustered by identity and shown under the various viewpoints side by side. All learned identities were randomly presented one-by-one in the second familiarization phase, each under one of the seven viewpoints. The face appeared in isolation for 1000 ms. Then the name associated with the face was added, and both name and face were shown together for another 2000 ms. Participants were then evaluated on their ability to name the learned so-called ‘old’ faces at various viewpoints (+75° to –75°, in steps of 25°). A trial started with a 500 ms fixation; then the stimulus was presented at screen center along with the name options (the five names of the ‘old’ faces of the same gender as the shown face), numbered from 1 to 5, at the top of the screen until participant response (maximum of 3000 ms). Participants responded by pressing the corresponding key (1–5). The fixation turned green in case of a correct response. When the response was incorrect, the fixation turned red. In both cases, the correct name appeared along with feedback. Feedback lasted for 500 ms. Familiarization and test were looped until naming accuracy reached 80%.

During the experimental old/new recognition task, faces were presented one by one at screen center, and participants were instructed to determine for each of them whether it was a face with which they had been a priori familiarized (‘old’ face) or not (‘new’ face). They answered using a button box by pressing ‘1’ for ‘old’ and ‘2’ for ‘new’. The main experiment was divided into 32 runs of 40 trials. A run started with recap screens for all learned identities under the seven viewpoints (from +75° to –75° in 25° steps) along with their associated name. In each main experiment trial, a face stimulus was presented centrally at a specified viewpoint and orientation range (from 0° to 157.5° in 22.5° steps). Viewpoint and orientation range varied randomly from one trial to the other. A trial started with a 1 s fixation; next, the face stimulus appeared until response or for 3 s maximum. At the end of a run, participants received feedback on their average accuracy. To avoid inducing a response bias, there were as many ‘old’ as ‘new’ trials, making the 10 ‘old’ faces twice as frequent as the 20 ‘new’ ones, which were each presented only once per condition. There were 40 trials per condition and a total of 1280 trials. Participants first practiced the main experimental task on 20 randomly selected trials with full spectrum stimuli. If they reached 80% accuracy, they could start the main experiment with the filtered stimuli. If practice accuracy was lower than 80%, participants were invited to run the familiarization (learning and test) again. Whenever accuracy in an experiment run dropped below 55% correct, and every third run regardless of performance, participants were presented with the recap screens again. All through the experiment, they were encouraged to respond as accurately and rapidly as possible.

The total experiment lasted for 1.5 hr on average, split into three testing sessions.

### Human data analysis

To prevent outlier responses from contaminating the results, we applied a log10 transformation on response latencies at the trial level and excluded trials with latencies at more than 2.5 times the SD above and below the individual mean in each participant. This procedure resulted in the exclusion of 1.81% of the trials on average.

In order to estimate the orientation dependence of human sensitivity to identity across views, we derived individual *d’* at each viewpoint and orientation. To do so, we determined hit and false alarm rates (Tanner and Birdsall, 1958) from the ‘old’/’new’ response in each participant, at each orientation and for each viewpoint. Following the log-linear rule (Hautus, 1995), we added a 0.5 correction to both before calculating the z scores. Performance at the 0° filter was duplicated to have circular filter values from 0° to 180°. As expected from previous studies (Dumont et al., 2024; Goffaux and Greenwood, 2016; Pachai et al., 2018), the *d’* plotted as a function of orientation in the frontal view condition (yaw = 0°) depicted a bell-shaped function centered on horizontal orientation (i.e. 90°; see Figure 3A, central panel). At the other viewpoints, *d’* followed a very similar shape, with maximum sensitivity roughly centered on horizontal orientation. Therefore, fitting the human sensitivity data using a Gaussian model seemed most appropriate as it allows characterizing the parameters of the tuning profile, namely, peak location, peak amplitude, standard deviation, and base amplitude, which are directly interpretable in cognitive/functional terms. Moreover, the use of a nonlinear, Gaussian model is motivated by past work that showed that the Gaussian function fits the evolution of recognition performance as a function of orientation (Dakin and Watt, 2009; Goffaux and Greenwood, 2016). Simpler frameworks, that is a linear model predicting *d’* from the interaction between orientation and viewpoint, would result in an 8 (orientation) * 7 (viewpoint) design that is difficult to analyze and interpret.

The Gaussian model is defined as:

$$\displaystyle f\left (orientation\right)=BaseAmplitude+PeakAmplitude.{\rm exp} \left (- \frac{\left (orientation- PeakLocation\right)^2}{2StandardDeviation^2}\right)$$

with *orientation* being the orientation of the filter, ranging from 0 to 180°. The model estimates four free parameters. *Peak location* is the orientation on which the Gaussian curve is centered. *Standard deviation* is the width of the Gaussian curve (i.e. strength of the tuning). *Base amplitude* is the height of the Gaussian curve base (i.e. the minimum sensitivity, typically found near vertical orientations). *Peak amplitude* refers to the height of the Gaussian curve relative to its baseline, reflecting the advantage of horizontal over vertical orientations for horizontally centered Gaussians.

We used the package *brms* (Bürkner, 2018) in R to implement this model in the Bayesian framework, using a multilevel modeling approach (Dumont et al., 2024; Moors et al., 2020; Nalborczyk et al., 2019). The four parameters *peak location*, *standard deviation*, *base amplitude*, and *peak amplitude* were conjointly estimated by linear predictor terms which included an intercept and the effect of Viewpoint. We also estimated a subject-level intercept of *standard deviation* and *base amplitude* as random effects, to allow the shape of the Gaussian to vary across participants. This multilevel structure provides a more accurate estimation of population-level parameters by accounting for subject variability. The prior distributions of the different parameters of the model were specified based on a compromise between (1) using knowledge from previous research (e.g. we used a normal distribution centered on horizontal orientation – 90° – for *peak location*, in line with the well-established horizontal tuning of face identification), (2) keeping unbiased uncertainty when previous research was not informative (e.g. for the effect of Viewpoint on the different parameters), and (3) allowing the convergence of the model. The details of the prior distributions can be found in Supplementary file 1. We ran four Markov Chain Monte-Carlo simulations, with 20,000 iterations including 3000 warm-up iterations.

Model diagnostics of the model were checked and indicated a good convergence: the potential scale reduction factor (R-hat) was of 1.00 for all parameters, the Bulk Effective Sample Size (ESS) was superior to 10,000 for all but four parameters (intercept of the base amplitude: Bulk ESS = 5504; intercept of the peak amplitude: Bulk ESS = 8494; subject-level random effect of the standard deviation: Bulk ESS = 5965; subject-level random effect of the base amplitude: Bulk ESS = 6308), and the Tail ESS was superior to 10,000 for all but one parameter (subject-level random effect of the standard deviation: Bulk ESS = 5321).

### Image analysis

We investigated how the distribution of energy across orientations in the experimental stimuli varied as a function of viewpoint. The amplitude and phase spectra for the image of each full spectrum face were obtained by means of a two-dimensional fast Fourier Transform and multiplied with wrapped Gaussian filters, with peak orientations centered on orientation values from 0° to 157.5° in steps of 22.5° (20° standard deviation; as in Goffaux and Greenwood, 2016) and with peak spatial frequencies ranging from 1 to 200 cycles per image in 20 logarithmic steps. Amplitude values within each spatial frequency and orientation bin were squared and summed, then averaged across spatial frequencies per orientation bin.

Note that because the Fourier transform represents image energy in a discrete manner, energy at the lowest spatial frequency components can only be reliably sampled at the main cardinal and oblique ranges (i.e. 0°, 45°, 90°, 135°) especially when very narrow orientation filters are used (Hansen and Essock, 2004). However, because orientation filters were broad, and we interpret relative differences and not absolute values of energy distribution across viewpoints, the influence of this issue is minimal. We obtained one mean energy value per orientation band by averaging across identities.

We found that while face images contained most of their energy in the horizontal range, irrespective of viewpoint (Figure 3), the amplitude of the horizontal energy peak decreased as the face viewpoint moved away from frontal. For profile views, there was a plateau covering the horizontal plus the adjacent left and right oblique orientations for the left- and right-pointing profile views, respectively. In comparison, vertical energy was lower than any other range regardless of viewpoint. These findings replicate past evidence that most of the energy in a face image is contained in a range centered over the horizontal angle (Goffaux, 2019; Goffaux and Greenwood, 2016; Keil, 2009).

Yet the distribution of image energy as a function of orientation does not provide any insight about its potential usefulness for face identity recognition; we addressed this using model observers.

### Model observers

To systematically quantify the orientation profile of the stimulus information physically available to match identity either (1) within a fixed viewpoint or (2) across distinct viewpoints (i.e. thus requiring the generalization of identity from one view to other views), we designed two different model observers: a view-selective model observer and a view-tolerant model observer, respectively. By characterizing the orientation profile of the information available to recognize face identity in a view-selective and -tolerant manner, this approach enables us to investigate objectively whether and how this information is used by humans (Collin et al., 2014; Pachai et al., 2013). Namely, it allows addressing whether the orientation range used during view-tolerant identity recognition by humans is the most informative for the view at stake, or the one most stable across views, at the physical image level.

Model observers matched the same randomly selected 10 ‘old’ faces and 20 ‘new’ faces as the human observers; we ran 22 view-selective and 22 view-tolerant model observers to match the number of tested human subjects. We presented each of the 30 faces once per condition. In each trial, the models computed the pixelwise similarity based on the calculation of a cross-correlation between an orientation-filtered target image (either from the ‘old’ or ‘new’ set) and each of the possible exemplars of the same gender (i.e. probes) filtered at the same orientation as the target image. The probe face with the highest correlation (i.e. the more similar) to the target image was selected as the model response (winner-take-all scheme). Depending on the correspondence between the ‘winner’ probe and the actual target, the responses of the model observer were categorized as hit or false alarm, allowing for the computation of a sensitivity *d’* in each orientation and viewpoint condition along a procedure like the one used to compute human performance.

In both model types, targets and probes were of the same orientation range (see Collin et al., 2014; Näsänen, 1999 for a similar method applied to the spatial frequency domain). Targets and probes in the view-selective model observer stemmed from a fixed viewpoint whereas the view-tolerant model observer matched target and probe separated by more than one viewpoint step to force tolerance to viewpoint in this model performance (e.g. a face at +025 of yaw was matched to faces at +075, –025, –050, and –075). This separation of views between target and probes was essential to force the view-tolerant model observer to generalize identity across views when matching identity at the image pixel level. It is not meant to be a computationally-valid analogy of how humans recognize identity in a view-tolerant manner. Since profile views stood at the viewpoint continuum extrema, one extra viewpoint was available for comparison. We therefore decided to drop the mirror profile view to match the number of comparisons across profile and non-profile viewpoints. Performance of the view-tolerant model was averaged across comparison viewpoints.

In a pilot phase, we measured the overall identification performance of each model. Initially, the view-selective model performed at ceiling, yielding a correlation of 1 since there was an exact target-probe match across all trials. To avoid ceiling effects and to keep model performance close to human levels (Figure 4—figure supplement 1), we thus decreased the signal-to-noise ratio (SNR) of the target and probe images to 0.125 by combining each with distinct noise patterns (face RMS contrast: 0.01; noise RMS contrast: 0.08). Each trial (i.e. target-probe pairing) was iterated ten times with different random noise patterns. Sensitivity *d’* of the view-tolerant model was much lower than view-selective model and human sensitivity (Figure 4—figure supplement 1), even without noise. The view-tolerant model therefore processed fully visible stimuli (SNR of 1). This decreased sensitivity in the view-tolerant compared to the view-selective model is expected, as none of the probes exactly matched the target at the pixel level due to viewpoint differences. In contrast to humans who rely on internally stored representations to match identity across views, the model observer lacks such internal representations and entirely relies on (less efficient) pixelwise comparisons.

The main objective of running these model observers is to interpret human orientation sensitivity profiles considering the available information in the stimulus; following this reasoning, model observer performance is only interesting when compared to human performance. Therefore, we investigated which model observer best predicted the orientation dependence profile of human face recognition using partial Pearson correlation analyses. We controlled for the variance in image energy across orientations and viewpoints in all partial correlation analyses to exclude the possibility that view-selective/tolerant model performance was a mere epiphenomenon of absolute oriented energy. The Fisher Z-transform of perfect correlations leading to infinite values, we replaced all –1 and 1 partial correlation coefficients to –0.99 and 0.99, respectively, before applying Fisher Z-transformation and computing 95% confidence intervals (see Table 1). We (partial) correlated the orientation profiles of human and each model observer at each viewpoint separately (eight orientation vectors). For each specific viewpoint, the orientation sensitivity profile of each human observer was correlated to the average orientation sensitivity profile of either model observer, while controlling for the variance explained by (1) the average orientation sensitivity profile of the alternate model observer and (2) the average profile of orientation energy. The variability in human individual orientation profiles was taken as an estimate of the maximally achievable data-model correlation. We computed the cross-correlation between each human individual orientation sensitivity profile and the average sensitivity profile of the remaining participants. The maximally achievable correlation was the mean of these individual-to-group correlations.

The codes used for stimulus generation and data analyses as well as the stimulus images, the experimental stimulation scripts, and all data (human and model observer) are publicly available at https://osf.io/fe8s9/.

## Data Availability

The stimuli and experimental stimulation scripts, the human and model observer data is publicly available at https://osf.io/fe8s9/. The following dataset was generated: Roux-SibilonA DumontH GoffauxV 2026Viewpoint & Orientation dependence of Face identificationOpen Science Frameworkfe8s9

## Additional files

Supplementary file 1. Prior distributions of the four parameters of the Gaussian Bayesian multilevel model.

MDAR checklist

## Appendix 1

### View-average model observer: methods and results

#### Methods

The building up of stable identity representations is thought to rely on some kind of average summary of the variable face appearance. We designed an additional model observer to test the hypothesis that horizontal face information may be better suited to match such average summary representation (Figures 1 and 6A in the Manuscript).

Akin to the view-selective and view-tolerant model observers, the view-average model observer performed the task on the same randomly selected 10 ‘old’ faces and 20 ‘new’ faces as the human observers. We ran 22 view-average model observers to match the number of tested human subjects. We presented each of the 30 faces once per condition. In each trial, the models computed the pixelwise similarity based on the calculation of a cross-correlation between a target image (either from the ‘old’ or ‘new’ set) and each of the possible exemplars of the same gender (i.e. probes) filtered at the same orientation as the target image. The probe face with the highest correlation (i.e. the more similar) to the target image was selected as the model response (winner-take-all scheme). Depending on the correspondence between the ‘winner’ probe and the actual target, the responses of the model observer were categorized as hit or false alarm, allowing for the computation of a sensitivity *d’* in each orientation and viewpoint condition along a procedure like the one used to compute human performance.

Importantly, targets were orientation-filtered faces seen under different viewpoints while probes were the averages of the full-spectrum images of the different views of each identity (Figure 6A in the Manuscript). In a pilot phase, we measured the overall identification performance of the model. To avoid ceiling effects and to keep model performance close to human levels (Figure 4—figure supplement 1), we decreased the signal-to-noise ratio (SNR) of the target and probe images to 0.5 (face RMS contrast: 0.04; noise RMS contrast: 0.08). In all trials, target and probe faces were each combined with 10 different random noise patterns.

Next, we investigated the extent to which this model observer predicted the orientation dependence profile of human face recognition using Pearson correlation analyses at each viewpoint separately. The Fisher Z-transform of perfect correlations leading to infinite values, we replaced all –1 and 1 partial correlation coefficients to –0.99 and 0.99, respectively, before applying Fisher Z-transformation and computing 95% confidence intervals. For each specific viewpoint, the orientation sensitivity profile of each human observer was correlated to the average orientation sensitivity profile of the view-average model observer. The variability in human individual orientation profiles was taken as an estimate of the maximally achievable data-model correlation. Specifically, we computed the cross-correlation between each human individual orientation sensitivity profile and the average sensitivity profile of the remaining participants. The maximally achievable correlation was the mean of these individual-to-group correlations.

#### Results

The view-average model performed best in the horizontal range, irrespective of yaw (Figure 6B in the Manuscript). In other words, the different views of faces were more accurately linked to the full-spectrum view average of the matching face identity when they were filtered to contain horizontally structured information than when they were filtered to contain the other orientations. Sensitivity decreased from horizontal towards vertical orientations at all views except for the two most extreme left views (+050 and+075). In these latter views, sensitivity reached its minimum in the oblique orientations and rebounded upward in the vertical range. Due to the stability of the horizontal sensitivity advantage of the view-average model observer, its orientation tuning profile was correlated across all views, but more strongly so between –075 and +025 views and between +050 and +075 views due to vertical sensitivity differences (Figure 6B in the Manuscript, rightmost map).
