# Supplementary material for: The view tolerance of human identity recognition depends on horizontal face information
Source: eLife. 2026 Jul 20;14:RP108495. doi: 10.7554/eLife.108495 (PMC13384493; doi:10.7554/eLife.108495)
Supplement: Supplementary file 1. [file elife-108495-supp1.docx]

# Supplementary File 1. Prior distributions of the four parameters of the Gaussian Bayesian multilevel model.

|  | Peak Location | Standard Deviation | Peak Amplitude | Base Amplitude |
| --- | --- | --- | --- | --- |
| Prior for Intercept | **normal(90, 3)** | **normal(40, 3)** | **normal(2.32, 1.5)** | **normal(2.32, 1.5)** |
| Prior for the slope (effect of Viewpoint) | Viewpoints -50, -25, 25, 50: **normal(0, 5)**  Viewpoints -75, 75: **normal(0, 10)** | **normal(0, 5)** | **normal(0, 1)** | **normal(0, 1)** |
| Prior for random effects | *NA* | **exponential(0.1)** | *NA* | **exponential(0.1)** |
