# Supplementary material for: The view tolerance of human identity recognition depends on horizontal face information
Source: eLife. 2026 Jul 20;14:RP108495. doi: 10.7554/eLife.108495 (PMC13384493; doi:10.7554/eLife.108495)
Supplement: Peer review history

# Editor report: eLife Assessment

**DOI:** 10.7554/eLife.108495.4.sa0

**Authors:** Tessa Dekker

**Affiliations:** University College London United Kingdom

**Keywords:** Convincing, Important

This **important** study combines behavioural psychophysics with image-based observer modelling to investigate which visual information can support view-tolerant face identity recognition. It offers **convincing** evidence that although diagnostic orientation content about identity varies with viewpoint - more horizontal for frontal views and more vertical for profiles - human recognition remains mainly tuned to horizontal information, identified by a view-tolerant model as carrying the most stable identity cues across viewpoints. Questions remain about how this generalises to ecological scenes and is biologically implemented.

# Referee report: Reviewer #1 (Public review)

**DOI:** 10.7554/eLife.108495.4.sa1

[Editors' note: this version has been assessed by the Reviewing Editor without further input from the original reviewers. The authors have addressed the comments raised in the previous round of review.]

Summary:

The authors describe the results of a single study designed to investigate the extent to which horizontal orientation energy plays a key role in supporting view-invariant face recognition. The authors collected behavioral data from adult observers who were asked to complete an old/new face matching task by learning broad-spectrum faces (not orientation filtered) during a familiarization phase and subsequently trying to label filtered faces as previously seen or novel at test. This data revealed a clear bias favoring the use of horizontal orientation energy across viewpoint changes in the target images. The authors then compared different ideal observer models (cross-correlations between target and probe stimuli) to examine how this profile might be reflected in the image-level appearance of their filtered images. This revealed that a model looking for the best matching face within a viewpoint differed substantially from human data, exhibiting a vertical orientation bias for extreme profiles. However, a model forced to match targets to probes at different viewing angles exhibited a consistent horizontal bias in much the same manner as human observers.

Strengths:

I think the question is an important one: The horizontal orientation bias is a great example of a low-level image property being linked to high-level recognition outcomes and understanding the nature of that connection is important. I found the old/new task to be a straightforward task that was implemented ably and that has the benefit of being simple for participants to carry out and simple to analyze. I particularly appreciated that the authors chose to describe human data via a lower-dimensional model (their Gaussian fits to individual data) for further analysis. This was a nice way to express the nature of the tuning function favoring horizontal orientation bias in a way that makes key parameters explicit. Broadly speaking, I also thought that the model comparison they include between the view-selective and view-tolerant models was a great next step. This analysis has the potential to reveal some good insights into how this bias emerges and ask fine-grained questions about the parameters in their model fits to the behavioral data.

Weaknesses:

I'll start with what I think is the biggest difficulty I had with the paper. Much as I liked the model comparison analysis, I also don't quite know what to make of the view-tolerant model. As I understand the authors' description, the key feature of this model is that it does not get to compare target and probe at the same yaw angle, but must instead pick a best match from candidates that are at different yaws. While it is interesting to see that this leads to a very different orientation profile, it also isn't obvious to me why such a comparison would be reflective of what the visual system is probably doing. I can see that the view-specific model is more or less assuming something like an exemplar representation of each face: You have the opportunity to compare a new image to a whole library of viewpoints and presumably it isn't hard to start with some kind of first pass that identifies the best matching view first before trying to identify/match the individual in question. What I don't get about the view-tolerant model is that it seems almost like an anti-exemplar model: You specifically lack the best viewpoint in the library but have to make do with the other options. I sort of understand the reasoning that this enforces tolerance of viewpoint variability, but I'm not clear on whether or not this is a version of face familiarity and recognition that the authors think has an analog in human visual processing.

I do think that this model is interesting in terms of the differential tuning it exhibits, but don't find it easy to align with any theoretical perspective on face recognition. Specifically, do the authors think there is a stage of face processing in which tolerance as they've operationalized it in the model is extant? What I'm looking for is a concrete description of the circumstances that the authors are saying lead to this kind of model potentially being a meaningful analog of face recognition. For example, is the idea that one may become familiar with a face in some very limited set of viewpoints and then be presented with that face in other views?

Alternatively, if the authors prefer to say that they simply thought this was a nice exercise in terms of identifying a different model and that it may not be a meaningful proxy for face recognition. I think that's fine, to be clear! I just still don't see anything in the text that convinces me of the ecological validity of this version of view-tolerance.

# Referee report: Reviewer #2 (Public review)

**DOI:** 10.7554/eLife.108495.4.sa2

This study investigates the visual information that is used for the recognition of faces. This is an important question in vision research and is critical for social interactions more generally. The authors ask whether our ability to recognise faces, across different viewpoints, varies as a function of the orientation information available in the image. Consistent with previous findings from this group and others, they find that horizontally filtered faces were recognised better than vertically filtered faces. Next, they probe the mechanism underlying this pattern of data by designing two model observers. The first was optimised for faces at a specific viewpoint (view-selective). The second was generalised across viewpoints (view-tolerant). In contrast to the human data, the view-specific model shows that the information that is useful for identity judgements varies according to viewpoint. For example, frontal face identities are again optimally discriminated with horizontal orientation information, but profiles are optimally discriminated with more vertical orientation information. These findings show human face recognition is biased toward horizontal orientation information, even though this may be suboptimal for the recognition of profile views of the face.

One issue in the design of this study was the lowering of the signal-to-noise ratio in the view-selective observer. This decision was taken to avoid ceiling effects. However, it is not clear how this affects the similarity with the human observers.

Another issue is the decision to normalise image energy across orientations and viewpoints. I can see the logic in wanting to control for these effects, but this does reflect natural variation in image properties. So, again, I wonder what the results would look like without this step.

Despite the bias toward horizontal orientations in human observers, there were some differences in the orientation preference at each viewpoint. For example, frontal faces were biased to horizontal (90 deg) but other viewpoints had biases that were slightly off horizontal (e.g. right profile: 80 deg, left profile: 100 deg). This does seem to show that differences in statistical information at different viewpoints (more horizontal information for frontal and more vertical information for profile) do influence human perception. It would be good to reflect on this nuance in the data.

Comments on revisions:

I am happy with the response and changes to the comments in my review. The key findings from this study are: (1) that there is bias toward the use of horizontal information across all viewpoints for face recognition in humans using an old-new recognition task. (2) In contrast, the optimal information for matching faces varies as a function of viewpoint. The view-selective model shows horizontal information is dominant for frontal views and vertical information is dominant for profile views.

The data from the view-tolerant model is less easy to interpret as it doesn't fit with any theoretically plausible model of face recognition. It might be a useful model for a face matching task in which participants had to match unfamiliar faces across viewpoints. This might be a possible extension of the current work.

Nonetheless, I still think this is an interesting contribution to the literature.

# Author comment: Author response

**DOI:** 10.7554/eLife.108495.4.sa3

**Authors:** Alexia Roux-Sibilon, Helene Dumont, Vincent Bremhorst, Christianne Jacobs, Valerie Goffaux

**Affiliations:** Université Clermont Auvergne Clermont-Ferrand France; UCLouvain Louvain-la-Neuve Belgium; UCLouvain Louvain-la-Neuve Belgium; UCLouvain Louvain-la-Neuve Belgium; UCLouvain Louvain-la-Neuve Belgium

The following is the authors’ response to the previous reviews

**Reviewer #1 (Public review):**

Summary:

The authors describe the results of a single study designed to investigate the extent to which horizontal orientation energy plays a key role in supporting view-invariant face recognition. The authors collected behavioral data from adult observers who were asked to complete an old/new face matching task by learning broad-spectrum faces (not orientation filtered) during a familiarization phase and subsequently trying to label filtered faces as previously seen or novel at test. This data revealed a clear bias favoring the use of horizontal orientation energy across viewpoint changes in the target images. The authors then compared different ideal observer models (cross-correlations between target and probe stimuli) to examine how this profile might be reflected in the image-level appearance of their filtered images. This revealed that a model looking for the best matching face within a viewpoint differed substantially from human data, exhibiting a vertical orientation bias for extreme profiles. However, a model forced to match targets to probes at different viewing angles exhibited a consistent horizontal bias in much the same manner as human observers.

Strengths:

I think the question is an important one: The horizontal orientation bias is a great example of a low-level image property being linked to high-level recognition outcomes, and understanding the nature of that connection is important. I found the old/new task to be a straightforward task that was implemented ably and that has the benefit of being simple for participants to carry out and simple to analyze. I particularly appreciated that the authors chose to describe human data via a lower-dimensional model (their Gaussian fits to individual data) for further analysis. This was a nice way to express the nature of the tuning function, favoring horizontal orientation bias in a way that makes key parameters explicit. Broadly speaking, I also thought that the model comparison they include between the view-selective and view-tolerant models was a great next step. This analysis has the potential to reveal some good insights into how this bias emerges and ask fine-grained questions about the parameters in their model fits to the behavioral data.

Weaknesses:

I will start with what I think is the biggest difficulty I had with the paper. Much as I liked the model comparison analysis, I also don't quite know what to make of the view-tolerant model. As I understand the authors' description, the key feature of this model is that it does not get to compare the target and probe at the same yaw angle, but must instead pick a best match from candidates that are at different yaws. While it is interesting to see that this leads to a very different orientation profile, it also isn't obvious to me why such a comparison would be reflective of what the visual system is probably doing. I can see that the view-specific model is more or less assuming something like an exemplar representation of each face: You have the opportunity to compare a new image to a whole library of viewpoints, and presumably it isn't hard to start with some kind of first pass that identifies the best matching view first before trying to identify/match the individual in question. What I don't get about the view-tolerant model is that it seems almost like an anti-exemplar model: You specifically lack the best viewpoint in the library but have to make do with the other options. Again, this is sort of interesting and the very different behavior of the model is neat to discuss, but it doesn't seem easy to align with any theoretical perspective on face recognition. My thinking here is that it might be useful to consider an additional alternate model that doesn't specifically exclude the best-matching viewpoint, but perhaps condenses appearance across views into something like a prototype. I could even see an argument for something like the yaw-averages presented earlier in the manuscript as the basis for such a model, but this might be too much of a stretch. Overall, what I'd like to see is some kind of alternate model that incorporates the existence of the best-match viewpoint somehow, but without the explicit exemplar structure of the view-specific model.

The design of the view-tolerant model aligned with the requirements of tolerant recognition and revealed the stimulus information enabling to abstract identity away from variations in face appearance. However, it did not involve the notion that such ability may depend on a prototype or summary representation of face identity built up through varied encounters (Burton, Jenkins, & Schweinberger, 2011; Burton et al., 2016; Jenkins et al., 2011; Menon, Kemp, & White, 2018; Mike Burton, 2013).

We agree with the Reviewer that the average of the different views of a face is a good proxy of its central tendency (i.e., stable identity properties; Figure 1). We thus followed their suggestion and included an additional model observer that compared specific views to full-spectrum view-averaged identities. The examination of the orientation tuning profile of this so-called view-average model observer confirmed the crucial contribution of horizontal identity cues to view-invariant recognition as the horizontal range best predicted the average summary of full-spectrum face appearances across views. This additional model observer is now presented in the Discussion and Supplementary files 2 and 3.

Besides this larger issue, I would also like to see some more details about the nature of the cross-correlation that is the basis for this model comparison. I mostly think I get what is happening, but I think the authors could expand more on the nature of their noise model to make more explicit what is happening before these cross-correlations are taken. I infer that there is a noise-addition step to get them off the ceiling, but I felt that I had to read between the lines a bit to determine this.

In the Methods section, we now provide detailed information about the addition of noise to model observer cross-correlations: ‘In a pilot phase, we measured the overall identification performance of each model. Initially, the view-selective model performed at ceiling, yielding a correlation of 1 since there was an exact target-probe match across all trials. To avoid ceiling effects and to keep model performance close to human levels (Supplementary File 2), we thus decreased the signal-to-noise ratio (SNR) of the target and probe images to .125 by combining each with distinct noise patterns (face RMS contrast: .01; noise RMS contrast: .08). Each trial (i.e. target-probe pairing) was iterated ten times with different random noise patterns.’

We also added a supplemental with the graphic illustration of the d’ distributions of each model and human observers: ‘Sensitivity d’ of the view-tolerant model was much lower than view-selective model and human sensitivity (Supplementary File 2), even without noise. The view-tolerant model therefore processed fully visible stimuli (SNR of 1). This decreased sensitivity in the view-tolerant compared to the view-selective model is expected, as none of the probes exactly matched the target at the pixel level due to viewpoint differences. In contrast to humans who rely on internally stored representations to match identity across views, the model observer lacks such internal representations and entirely relies on (less efficient) pixelwise comparisons.’

Another thing that I think is worth considering and commenting on is the stimuli themselves and the extent to which this may limit the outcomes of their behavioral task. The use of the 3D laser-scanned faces has some obvious advantages, but also (I think) removes the possibility for pigmentation to contribute to recognition, removes the contribution of varying illumination and expression to appearance variability, and perhaps presents observers with more homogeneous faces than one typically has to worry about. I don't think these negate the current results, but I'd like the authors to expand on their discussion of these factors, particularly pigmentation. Naively, surface color and texture seem like they could offer diagnostic cues to identity that don't rely so critically on horizontal orientations, so removing these may mean that horizontal bias is particularly evident when face shape is the critical cue for recognition.

Our stimuli were originally designed by Troje and Bulthoff (1996). These are 3D laser scans of white individuals aged between 20 and 40 years, posing with a neutral expression. Different views of the faces were shot under a fixed illumination. Ears and a small portion of the neck were visible while the hair region was removed. All face images had a normalized skin color and we further converted them to grayscales

While we agree that this stimulus set offers a restricted range of within- and between-identity variations compared to what is experienced in natural settings, we believe that the present findings generalize to more ecological viewing conditions. Indeed, past evidence showed that the recognition of face pictures shot under largely variable pose, age, expression, illumination, hair style is tuned to the horizontal range of the face stimulus (Dakin & Watt, 2009; Dumont, Roux-Sibilon, & Goffaux, 2024). In other words, our finding that view-tolerant identity recognition is mainly driven by horizontal face information would likely replicate with the use of a more ecological stimulus set.

Moreover, the skin color normalization and grayscale conversion, while limiting the range of face variability, did not eliminate the contribution of surface pigmentation in our study. It is thus unlikely that our findings exclusively reflect the orientation dependence of face shape processing. Pigmentation refers to all surface reflectance properties (Russell et al., 2006) and hue (color) is only one among others. The grayscaled 3D laser scanned faces used here contained natural variations in crucial surface cues such as skin albedo (i.e., how light or dark the surface appears) and texture (i.e., spatial variation in how light is reflected); they have actually been used to disentangle the role of shape and surface cues to identity recognition (e.g., Jiang et al., 2009; Russell et al., 2007; Russell et al., 2006; Troje & Bulthoff, 1996; Vuong et al., 2005). Moreover, a past study of ours demonstrated that the diagnosticity of the horizontal range of face information is not restricted to face shape cues; the specialized processing of face shape and surface both selectively rely on horizontal information (Dumont, Roux-Sibilon, & Goffaux, 2024).

For these reasons, the present findings are unlikely to be fully determined by shape processing, and we expect them to generalize to more ecological stimulus sets. We discuss these aspects in the revised manuscript.

**Reviewer #2 (Public review):**

This study investigates the visual information that is used for the recognition of faces. This is an important question in vision research and is critical for social interactions more generally. The authors ask whether our ability to recognise faces, across different viewpoints, varies as a function of the orientation information available in the image. Consistent with previous findings from this group and others, they find that horizontally filtered faces were recognised better than vertically filtered faces. Next, they probe the mechanism underlying this pattern of data by designing two model observers. The first was optimised for faces at a specific viewpoint (view-selective). The second was generalised across viewpoints (view-tolerant). In contrast to the human data, the view-specific model shows that the information that is useful for identity judgements varies according to viewpoint. For example, frontal face identities are again optimally discriminated with horizontal orientation information, but profiles are optimally discriminated with more vertical orientation information. These findings show human face recognition is biased toward horizontal orientation information, even though this may be suboptimal for the recognition of profile views of the face.

One issue in the design of this study was the lowering of the signal-to-noise ratio in the view-selective observer. This decision was taken to avoid ceiling effects. However, it is not clear how this affects the similarity with the human observers.

In the Methods section, we now provide detailed information about the addition of noise to model observer cross-correlations: ‘In a pilot phase, we measured the overall identification performance of each model. Initially, the view-selective model performed at ceiling, yielding a correlation of 1 since there was an exact target-probe match across all trials. To avoid ceiling effects and to keep model performance close to human levels (Supplementary File 2), we thus decreased the signal-to-noise ratio (SNR) of the target and probe images to .125 by combining each with distinct noise patterns (face RMS contrast: .01; noise RMS contrast: .08). Each trial (i.e. target-probe pairing) was iterated ten times with different random noise patterns.’

We also added a supplemental with the graphic illustration of the d’ distributions of each model and human observers.

Another issue is the decision to normalise image energy across orientations and viewpoints. I can see the logic in wanting to control for these effects, but this does reflect natural variation in image properties. So, again, I wonder what the results would look like without this step.

All stimuli were matched for luminance and contrast. It is crucial to normalize image energy across orientations as natural image energy is disproportionately distributed across orientations (e.g., Hansen et al., 2003). Images of faces cropped from their background as used here contain most of their energy in the horizontal range (Goffaux & Greenwood, 2016; Keil, 2008, 2009). If not normalized after orientation filtering, such uneven distribution of energy would boost recognition performance in the horizontal range across views. Normalization was performed across our experimental conditions merely to avoid energy from explaining the influence of viewpoint on the orientation tuning profile.

We were not aware of any systematic natural variations of energy across face views. To address this, we measured face average energy (i.e., RMS contrast) in the original stimulus set, i.e., before the application of any image processing or manipulation. Background pixels were excluded from these image analyses. Across yaws, we found energy to range between .11 and .14 on a 0 to 1 grayscale. This is moderate compared to the range of energy variations we measured across identities (from .08 to .18). This suggests that variations in energy across viewpoints are moderate compared to variations related to identity. It is unclear whether these observations are specific to our stimulus set or whether they are generalizable to faces we encounter in everyday life. They, however, indicate that RMS contrast did not substantially vary across views in the present study and suggest that RMS normalization is unlikely to have affected the influence of viewpoint on recognition performance.

In the revised methods section, we explicitly motivate energy normalization: ‘Images of faces cropped from their background as used here contain most of their energy in the horizontal range (Goffaux, 2019; Goffaux & Greenwood, 2016; Keil, 2009). Across yaws, we found face energy to range between .11 and .14 on a 0 to 1 grayscale, which is moderate compared to the range of face energy variations we measured across identities (from .08 to .18). To prevent energy from explaining our results, in all images, the luminance and RMS contrast of the face pixels were fixed to 0.55 and 0.15, respectively, and background pixels were uniformly set to 0.55. The percentage of clipped pixel values (below 0 or above 1) per image did not exceed 3%.’.

Despite the bias toward horizontal orientations in human observers, there were some differences in the orientation preference at each viewpoint. For example, frontal faces were biased to horizontal (90 degrees), but other viewpoints had biases that were slightly off horizontal (e.g., right profile: 80 degrees, left profile: 100 degrees). This does seem to show that differences in statistical information at different viewpoints (more horizontal information for frontal and more vertical information for profile) do influence human perception. It would be good to reflect on this nuance in the data.

Indeed, human performance data indicates that while identity recognition remains tuned to horizontal information, horizontal tuning peak shows some variation across viewpoints. We primarily focused on the first aspect because of its direct relevance to our research objective, but also discussed the second aspect: with yaw rotation, certain non-horizontal morphological features such as the jaw line or nose bridge, etc. may increasingly contribute to identity recognition, whereas at frontal or near frontal views, features are mostly horizontally-oriented (e.g., Keil, 2008, 2009). In the revised Discussion, we directly relate the modest fluctuations of peak location to yaw differences in face feature appearance.

**Recommendations for the authors:**

**Reviewing Editor Comments:**

Based on a discussion with the reviewers, we integrated the recommendations and reached a consensus on the eLife assessment. To move from a "solid" to a "compelling/convincing" strength-of-evidence rating, please address the reviewers' comments. Key points are to clarify and test the plausibility of the models (e.g., effects of different noise-addition steps, inclusion/exclusion of specific orientation channels in the view-dependent comparison, and alternative decision criteria), and to address or discuss the limitations of the stimulus set in capturing recognition under more naturalistic scenarios, for example, including texture cues.

**Reviewer #1 (Recommendations for the authors):**

I generally found the paper to be very well-written, so I have only a few minor comments here.

(1) I didn't really follow why the estimation of the Gaussian functions described in the text was preferred over a simpler ML framework. Do these approaches differ that much? I see references to prior studies in which these were applied, so I can certainly go check these out, but I could see value in adding just a bit of text to briefly make the case that this is important.

Employing a simpler linear framework, i.e. a linear model predicting d’ from the interaction between orientation and viewpoint, would result in an 8 (orientation) * 7 (viewpoint) design that is difficult to analyze. The interaction term would almost certainly reach significance but its interpretation would be limited. We would either have to rely on numerous local comparisons, which are not particularly informative for our research objectives (e.g., knowing whether d’ differs significantly between two adjacent orientations at a given viewpoint is of little relevance), or to use a polynomial contrast approach (testing the linear, quadratic, … up to the 7th order trends), which would also be difficult to interpret. For such complex, approximately Gaussian-shaped data, the highest-order polynomial trend would likely provide the best fit, but without offering meaningful insight.

In contrast, a nonlinear approach appears more appropriate. The Gaussian model we used allows us to characterize the parameters of the tuning profile, namely, peak location, peak amplitude, standard deviation (or bandwidth) and base amplitude. These parameters are not merely statistical parameters. Rather, they are directly interpretable in cognitive/functional terms. The peak location corresponds to the orientation at which the Gaussian curve is centred, i.e. the preferred orientation band for identity recognition. The standard deviation represents the width of the curve, reflecting the strength or selectivity of the tuning. The base amplitude is the height of the Gaussian curve base, indicating the minimum level of sensitivity, typically found near vertical orientation. Finally, the peak amplitude refers to the height of the Gaussian curve relative to its baseline, that is, it captures the advantage of horizontal over vertical orientations.

Moreover, the use of a nonlinear, Gaussian model is motivated by past work that showed that the Gaussian function fits the evolution of recognition performance as a function of orientation (Dakin & Watt, 2009; Goffaux & Greenwood, 2016). Orientation selectivity at primary stages of visual processing has also been modelled using Gaussian (or Difference of Gaussians; Ringach, Hawken, & Shapley, 2003).

We revised the data analysis section to include a justification for our use of a Gaussian model: “Therefore, fitting the human sensitivity data could be fitted using a simple Gaussian model. seemed most appropriate as it allows characterizing the parameters of the tuning profile, namely, peak location, peak amplitude, standard deviation and base amplitude, which are directly interpretable in cognitive/functional terms. Moreover, the use of a nonlinear, Gaussian model is motivated by past work that showed that the Gaussian function fits the evolution of recognition performance as a function of orientation (Dakin & Watt, 2009; Goffaux & Greenwood, 2016). Simpler frameworks, i.e. a linear model predicting d’ from the interaction between orientation and viewpoint, would result in an 8 (orientation) * 7 (viewpoint) design that is difficult to analyze and interpret.”

(2) When reporting the luminance and contrast of your stimuli, please make clear what these units and measures are. This was a case where I had to take a second to assure myself that I knew what the values meant.

We clarified that the luminance and contrast values reported in the manuscript are on a grey scale ranging from 0 to 1.

(3) In your Procedure section, I think describing the familiarization task right away would help the text flow more clearly. At present, you began talking about the old/new task, and I was immediately wondering how familiarization worked!

The procedure section now starts with the description of the familiarization task.

(4) p. 3 - "Culminates" doesn't seem like the right word here.

We agree and rephrased this way: ‘The tolerance of face identity recognition is stronger for familiar than unfamiliar faces’.

(5) p. 5 - I think "with the multiple" shouldn't have "the".

Indeed, we removed the “the”.

**Reviewer #2 (Recommendations for the authors):**

I enjoyed reading the manuscript, but thought the Introduction was a bit long. I wasn't sure about the relevance of the section on temporal contiguity. I think this might have been more relevant if this had been a manipulation in the design. So, I wonder if this might be shortened or removed to focus on the key questions. On the other hand, I found the overview of the view-selective and view-tolerant to be a bit brief. There is plenty of detail here, but I found it difficult to break down what was done when I first read it. It might be good to provide an overview in the Discussion too.

While past research on the contribution of temporal contiguity to face identity recognition brings interesting insights into the nature of the visual experience leading to view-tolerant performance, we agree with the Reviewer that this aspect is not directly at stake here. We reduced the review of this literature in the Introduction.

We clarified the description of the model observers as suggested by the reviewer and made sure to provide an overview of the model observers in the Discussion as well.

References.

Burton, A. M., Jenkins, R., & Schweinberger, S. R. (2011). Mental representations of familiar faces. Br J Psychol, 102(4), 943-958. https://doi.org/10.1111/j.2044-8295.2011.02039.x

Burton, A. M., Kramer, R. S., Ritchie, K. L., & Jenkins, R. (2016). Identity From Variation: Representations of Faces Derived From Multiple Instances. Cogn Sci, 40(1), 202-223. https://doi.org/10.1111/cogs.12231

Collin, C. A., Rainville, S., Watier, N., & Boutet, I. (2014). Configural and featural discriminations use the same spatial frequencies: a model observer versus human observer analysis. Perception, 43(6), 509-526. https://doi.org/10.1068/p7531

Dakin, S. C., & Watt, R. J. (2009). Biological "bar codes" in human faces. J Vis, 9(4), 2 1-10. https://doi.org/10.1167/9.4.2

Dumont, H., Roux-Sibilon, A., & Goffaux, V. (2024). Horizontal face information is the main gateway to the shape and surface cues to familiar face identity. PLOS ONE, 19(10), e0311225. https://doi.org/10.1371/journal.pone.0311225

Goffaux, V., & Greenwood, J. A. (2016). The orientation selectivity of face identification [Article de recherche] [peer-reviewed]. Scientific Reports, 6(34204), 34204. https://doi.org/10.1038/srep34204

Gold, J., Bennett, P. J., & Sekuler, A. B. (1999). Identification of band-pass filtered letters and faces by human and ideal observers. Vision Research, 39(21), 3537-3560. https://doi.org/10.1016/s0042-6989(99)00080-2

Hansen, B. C., Essock, E. A., Zheng, Y., & DeFord, J. K. (2003). Perceptual anisotropies in visual processing and their relation to natural image statistics. Network, 14(3), 501-526. http://www.ncbi.nlm.nih.gov/pubmed/12938769

Jenkins, R., White, D., Van Montfort, X., & Mike Burton, A. (2011). Variability in photos of the same face. Cognition, 121(3), 313-323. https://doi.org/10.1016/j.cognition.2011.08.001

Jiang, F., Dricot, L., Blanz, V., Goebel, R., & Rossion, B. (2009). Neural correlates of shape and surface reflectance information in individual faces. Neuroscience, 163(4), 1078-1091. https://doi.org/10.1016/j.neuroscience.2009.07.062

Keil, M. S. (2008). Does face image statistics predict a preferred spatial frequency for human face processing? Proc Biol Sci, 275(1647), 2095-2100. https://doi.org/10.1098/rspb.2008.0486

Keil, M. S. (2009). "I look in your eyes, honey": internal face features induce spatial frequency preference for human face processing. PLoS Comput Biol, 5(3), e1000329. https://doi.org/10.1371/journal.pcbi.1000329

Menon, N., Kemp, R. I., & White, D. (2018). More than a sum of parts: robust face recognition by integrating variation. R Soc Open Sci, 5(5), 172381. https://doi.org/10.1098/rsos.172381

Mike Burton, A. (2013). Why has research in face recognition progressed so slowly? The importance of variability. Quarterly journal of experimental psychology, 66(8), 1467-1485. https://doi.org/10.1080/17470218.2013.800125

Näsänen, R. (1999). Spatial frequency bandwidth used in the recognition of facial images. Vision Research, 39(23), 3824-3833. https://doi.org/10.1016/s0042-6989(99)00096-6

Oruc, I., Shafai, F., Murthy, S., Lages, P., & Ton, T. (2019). The adult face-diet: A naturalistic observation study. Vision Res, 157, 222-229. https://doi.org/10.1016/j.visres.2018.01.001

Ringach, D. L., Hawken, M. J., & Shapley, R. (2003). Dynamics of orientation tuning in macaque V1: the role of global and tuned suppression [Research Support, Non-U.S. Gov't Research Support, U.S. Gov't, P.H.S.]. Journal of neurophysiology, 90(1), 342-352. https://doi.org/10.1152/jn.01018.2002

Russell, R., Biederman, I., Nederhouser, M., & Sinha, P. (2007). The utility of surface reflectance for the recognition of upright and inverted faces. Vision Res, 47(2), 157-165. https://doi.org/10.1016/j.visres.2006.11.002

Russell, R., Sinha, P., Biederman, I., & Nederhouser, M. (2006). Is pigmentation important for face recognition? Evidence from contrast negation. Perception, 35(6), 749-759. https://doi.org/10.1068/p5490

Troje, N. F., & Bulthoff, H. H. (1996). Face recognition under varying poses: the role of texture and shape. Vision Res, 36(12), 1761-1771. https://doi.org/10.1016/0042-6989(95)00230-8

Vuong, Q. C., Peissig, J. J., Harrison, M. C., & Tarr, M. J. (2005). The role of surface pigmentation for recognition revealed by contrast reversal in faces and Greebles. Vision Res, 45(10), 1213-1223. https://doi.org/10.1016/j.visres.2004.11.015
